# DNA methylation maintenance at the p53 locus initiates biliary-mediated liver regeneration

**DOI:** 10.1038/s41536-022-00217-8

**Published:** 2022-03-29

**Authors:** Jianbo He, Yang Zhou, Chuanfang Qian, Danyang Wang, Zhuolin Yang, Zhuofu Huang, Junhui Sun, Rui Ni, Qifen Yang, Jingying Chen, Lingfei Luo

**Affiliations:** 1 Institute of Developmental Biology and Regenerative Medicine, Southwest University, Beibei, 400715 Chongqing, China; 2 University of Chinese Academy of Sciences (Chongqing), Chongqing Institute of Green and Intelligent Technology, Chinese Academy of Sciences, Beibei, 400714 Chongqing, China

**Keywords:** DNA methylation, Hepatotoxicity, Transdifferentiation

## Abstract

In cases of extensive liver injury, biliary epithelial cells (BECs) dedifferentiate into bipotential progenitor cells (BPPCs), then redifferentiate into hepatocytes and BECs to accomplish liver regeneration. Whether epigenetic regulations, particularly DNA methylation maintenance enzymes, play a role in this biliary-mediated liver regeneration remains unknown. Here we show that in response to extensive hepatocyte damages, expression of *dnmt1* is upregulated in BECs to methylate DNA at the *p53* locus, which represses *p53* transcription, and in turn, derepresses mTORC1 signaling to activate BEC dedifferentiation. After BEC dedifferentiation and BPPC formation, DNA methylation at the *p53* locus maintains in BPPCs to continue blocking *p53* transcription, which derepresses Bmp signaling to induce BPPC redifferentiation. Thus, this study reveals promotive roles and mechanisms of DNA methylation at the *p53* locus in both dedifferentiation and redifferentiation stages of biliary-mediated liver regeneration, implicating DNA methylation and *p53* as potential targets to stimulate regeneration after extensive liver injury.

## Introduction

The liver is the largest digestive organ and performs multiple physiological functions, such as metabolism and detoxification, with remarkable regenerative capacity. The hepatic parenchyma cells, including hepatocytes and cholangiocytes, enlarge and proliferate for restoring hepatic mass upon mild liver injury after partial hepatectomy (PH) and most of the drug-induced liver injury^1^. In case of severe liver injury, such as end-stage liver diseases, when the proliferation of existing hepatocytes is compromised, the only treatment is liver transplantation, but the donors are limited. Using zebrafish as liver injury model, we and others found that the biliary epithelial cells (BECs) dedifferentiate into bipotential progenitor cells (BPPCs) expressing the endoderm and hepatoblast markers and then redifferentiate into hepatocytes and BECs after extensive hepatocyte damages^2–4^. This injury-induced BECs-hepatocytes trans-differentiation in zebrafish is conserved in mammalian mice when blocking hepatocyte proliferation or long-term chronic liver injury^5–9^. Promoting the trans-differentiation from BECs into hepatocytes may have potential clinical significance for patients with severe liver diseases.

The zebrafish^2–4,10,11^ and mouse^5,6,9^ are widely used to explore the cellular and molecular mechanisms that govern the BECs to hepatocytes trans-differentiation. In the initiation stage of liver regeneration, BECs lose their identity and dedifferentiate into BPPCs with activation of endoderm and hepatoblast markers after severe liver injury^2,3,8,10^. mTOR complex 1 (mTORC1) has been reported to regulate the BECs dedifferentiation^12,13^. Furthermore, mTORC1 and Yap signaling can promote BECs expansion during the ductular reaction^14^. After activation, the BPPC proliferate and redifferentiate into mature hepatocytes and BECs to accomplish liver regeneration. The number of liver progenitors correlates with the severity of liver disease after inefficient differentiation^15^. These undifferentiated liver progenitors secrete inflammatory factors to exacerbate fibrosis and liver cancer^16,17^. Then, promoting BPPC redifferentiation will accelerate the BECs-mediated liver regeneration. Several canonical signaling pathways are involved in the process of redifferentiation from BPPCs to hepatocytes and BECs, such as Bmp, Notch, PI3K, EGFR, Wnt, Jak-Stat3, and FXR signaling^2–4,10,13,18–22^. Through high-throughput screens, the roles of critical factors, such as BET proteins, Hdac1, Kdm1, TET1, and Sox9b, in BECs-mediated liver regeneration have been uncovered^3,19,23–25^. However, the detailed molecular mechanisms by which the dedifferentiation from BECs into BPPCs remain largely unknown.

DNA methylation is an essential epigenetic mechanism regulating gene expression and organ regeneration^26^. Three enzymatically DNA methyltransferases, including DNMT1, DNMT3A, DNMT3B, have been found in the vertebrate. Whereas DNMT3A and DNMT3B are essential for de novo DNA methylation^27^, DNMT1 is the primary enzyme responsible for maintaining DNA methylation during cell division^28^. Importantly, 5-azacytidine (5azaC), a specific inhibitor of DNMT1, has been widely used to inhibit DNA methylation^29^. Several studies show that DNA methylation is involved in organ regeneration, including pancreas regeneration^30^, axonal regeneration^31,32^, and retina regeneration^33^. Liver-specific *Dnmt1* knockout in mice demonstrated that DNMT1 plays an indispensable role in the genomic stability of hepatocytes in growth and the cell survival of liver progenitors^34^. Besides, liver-specific *Uhrf1* deletion induces DNA hypomethylation, activates the pro-regenerative genes, and enhances liver regeneration after PH^35^. Furthermore, DNA demethylation caused by TET1 licenses adult cholangiocytes for organoid formation^24^. Although the roles of DNA demethylation have been reported in different liver injury models, the functions of DNMT1 and DNA methylation maintenance in biliary-mediated liver regeneration have not been investigated.

In this study, we explored the roles of DNA methylation maintenance by Dnmt1 in BECs-mediated liver regeneration. We used DNA methylation inhibitor and *dnmt1* mutant to address the BEC dedifferentiation and BPPC redifferentiation. We uncovered that the maintenance of DNA methylation at the *p53* locus promotes the BEC dedifferentiation through derepressing mTORC1 signaling and induces BPPC redifferentiation through derepressing Bmp signaling. Loss of *dnmt1* blocks liver regeneration. Furthermore, DNA methylation level is maintained in hepatic progenitor cells in mice fed with a CDE diet.

## Results

### The maintenance of DNA methylation is required for the BEC-mediated liver regeneration

**Fig. 1:**
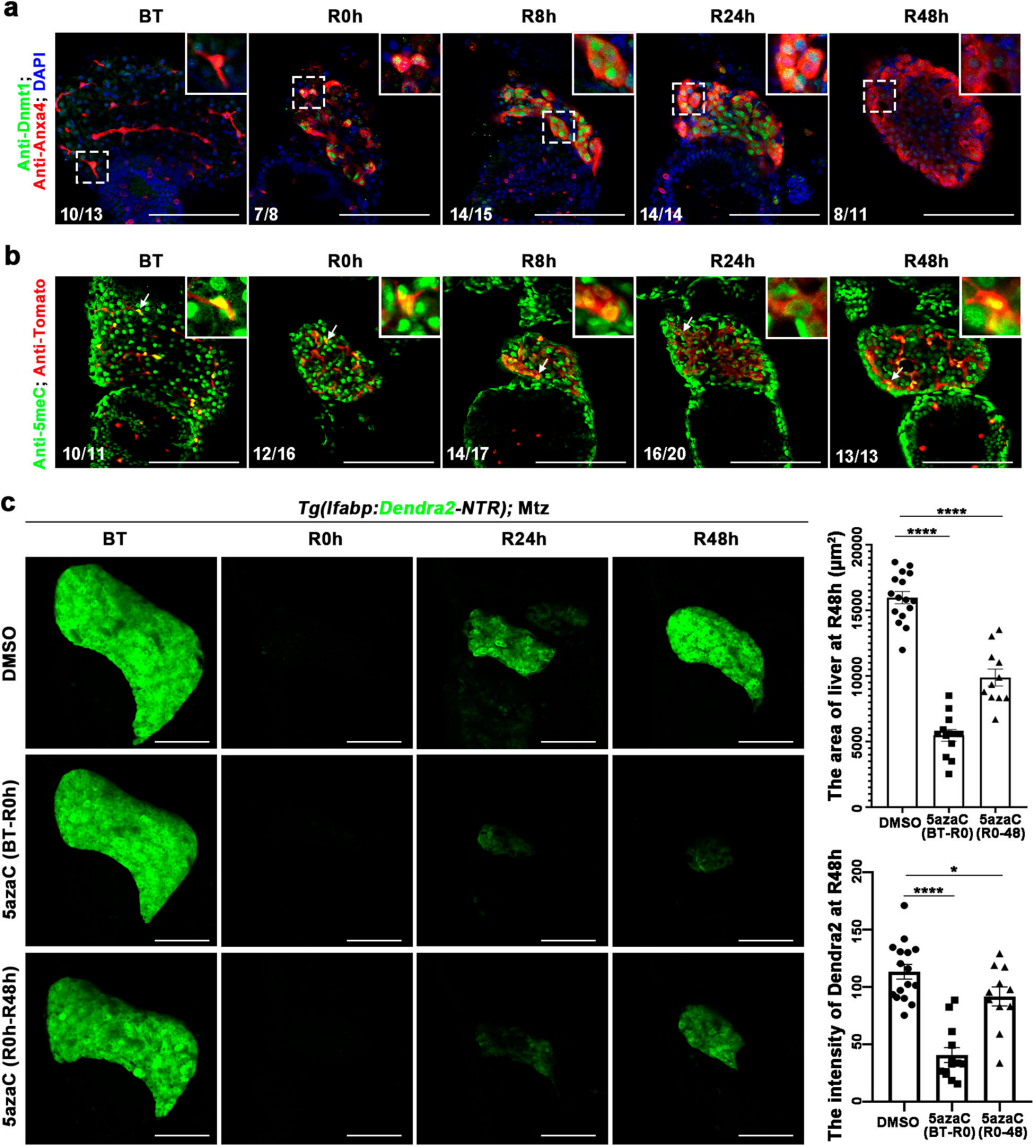
DNA methylation governs BECs-mediated liver regeneration. **a** Single-optical section images showing the expressions of Anxa4 and Dnmt1 in regeneration livers of *Tg(lfabp:Dendra2-NTR)* from BT to R48h. Note that the expression level of Dnmt1 was upregulated in Anxa4+ cells from R0h to R24h. **b** Single-optical section images showing the expressions of 5meC and Tomato in *Tg(lfabp:Dendra2-NTR; tp1:Tomato)* double transgenic line from BT to R48h. Note that the expression of 5meC maintains in Tomato+ cells from R0h to R24h (arrows). **c** Confocal projection images showing the liver regeneration from BT to R48h after 5azaC treatment from BT to R0h (early DNA methylation inhibition) and R0h to R48h (late DNA methylation inhibition). Quantification of the area of liver sizes and the intensity of Dendra2 expression at R48h. Asterisks indicate statistical significance: *P < 0.05; ****P < 0.0001 using *t*-tests analysis when compared to control. Numbers indicate the proportion of larvae exhibiting the expression shown. Scale bars: 100 µm; error bars, ±SEM. DAPI 4’, 6-diamidino-2-phenylindole, BT before treatment, R regeneration time after the withdrawal of Mtz.

Dnmt1 maintains DNA methylation during cell division and proliferation^28^. However, the roles of DNA methylation in liver regeneration after severe hepatocyte damages are unknown. To explore the roles of DNA methylation in liver regeneration after extensive hepatocyte loss, we applied the Nitroreductase-Metronidazole (NTR-Mtz)-based zebrafish liver injury model^2,3^ and detected the expressions of DNA methyltransferases, including *dnmt1*, *dnmt3aa*, *dnmt3ab*, and *dnmt3bb.1* (Supplementary Fig. 1). The expression of *dnmt1*, a DNA methylation maintenance enzyme, was specifically upregulated in the regenerating livers from regeneration 0 h (R0h) to R24h (Supplementary Fig. 1a). While the DNA de novo methylation enzymes, such as *dnmt3aa*, *dnmt3ab*, and *dnmt3bb.1*, were not expressed during liver regeneration (Supplementary Fig. 1b–1d). Anxa4, a BEC-specific marker^36^, labels BECs and the BECs-derived bipotential progenitor cells (BPPCs) during liver regeneration^2,3,12^. Whole-mount antibody staining further validated the expression of Dnmt1 specifically in the Anxa4-positive BPPCs from R0h to R24h and weak expression at R48h (Fig. 1a). To study DNA methylation levels during liver regeneration, we analyzed the expression of 5-methyl Cytosine (5meC), an indicator of DNA methylation^37^. In the *Tg(tp1:Tomato; lfabp:Dendra2-NTR)* transgenic line in which the BECs and BPPCs were labeled by Tomato, the expression of 5meC maintained in the Tomato-positive BPPCs from R0h to R48h (Fig. 1b, arrows). To explore the roles of Dnmt1 and DNA methylation in liver regeneration, 5‐azacytidine (5azaC), a specific inhibitor of Dnmt1^29^, was applied at different stages of liver regeneration (Fig. 1c and Supplementary Fig. 2a). The 5azaC treatment significantly reduced the expression levels of 5meC in BPPCs (Supplementary Fig. 2b). The larvae treated with 10 mM Mtz from 5 days post-fertilization (dpf) (before treatment, BT) to 6 dpf for 24 h regenerated normal liver with functional hepatic markers ceruloplasmin (*cp*) and vitamin D binding protein (*gc*)^2,3,12^ at R48h (Fig. 1c and Supplementary Fig. 2c). Consequently, after 5azaC treatment from 5 dpf/BT to 6 dpf/R0h (early inhibition of DNA methylation), liver regeneration hardly occurred, as exhibited by the Dendra2 epifluoscence or expressions of functional hepatocyte markers *cp* and *gc*^2,3,12^ (Fig. 1c and Supplementary Fig. 2c). While 5azaC treatment from R0h to R48h (late inhibition of DNA methylation) led to small regenerating livers (Fig. 1c and Supplementary Fig. 2c). The morphology of larvae remained normal after 5azaC treatment, excluding any side effects on liver regeneration (Supplementary Fig. 2d). These data indicate that the maintenance of DNA methylation plays important roles in liver regeneration.

To further confirm whether newly regenerated hepatocytes come from BEC trans-differentiation, we first analyzed hepatocyte markers *bhmt*, *fabp10a*, and *ttr* (Supplementary Fig. 3a). At R0h after Mtz treatment, hepatocytes were nearly complete loss (Fig. 1c), and hepatocyte markers became undetectable by whole-mount in situ hybridization (WISH) (Supplementary Fig. 3a). Then, the Cre/loxP-mediated genetic lineage tracing was carried out using the *Tg(krt18:CreER)* transgenic line with inducible Cre recombinase driven by the *krt18* promoter^12^. Validated by the *Tg(krt18:CreER; βactin:loxP-DsRed-loxP-GFP)* (Supplementary Fig. 3b), nearly all the newly regenerated hepatocytes came from the trans-differentiation of BECs in the *Tg(krt18:CreER; lfabp:loxP-STOP-loxP-DsRed; lfabp:Dendra2-NTR)* line after 5azaC treatment (Supplementary Fig. 3c and 3d). These data suggest that the 5azaC treatment does not affect the origins of nascent hepatocytes.

### DNA methylation maintenance governs BEC dedifferentiation at the early stage of liver regeneration

**Fig. 2:**
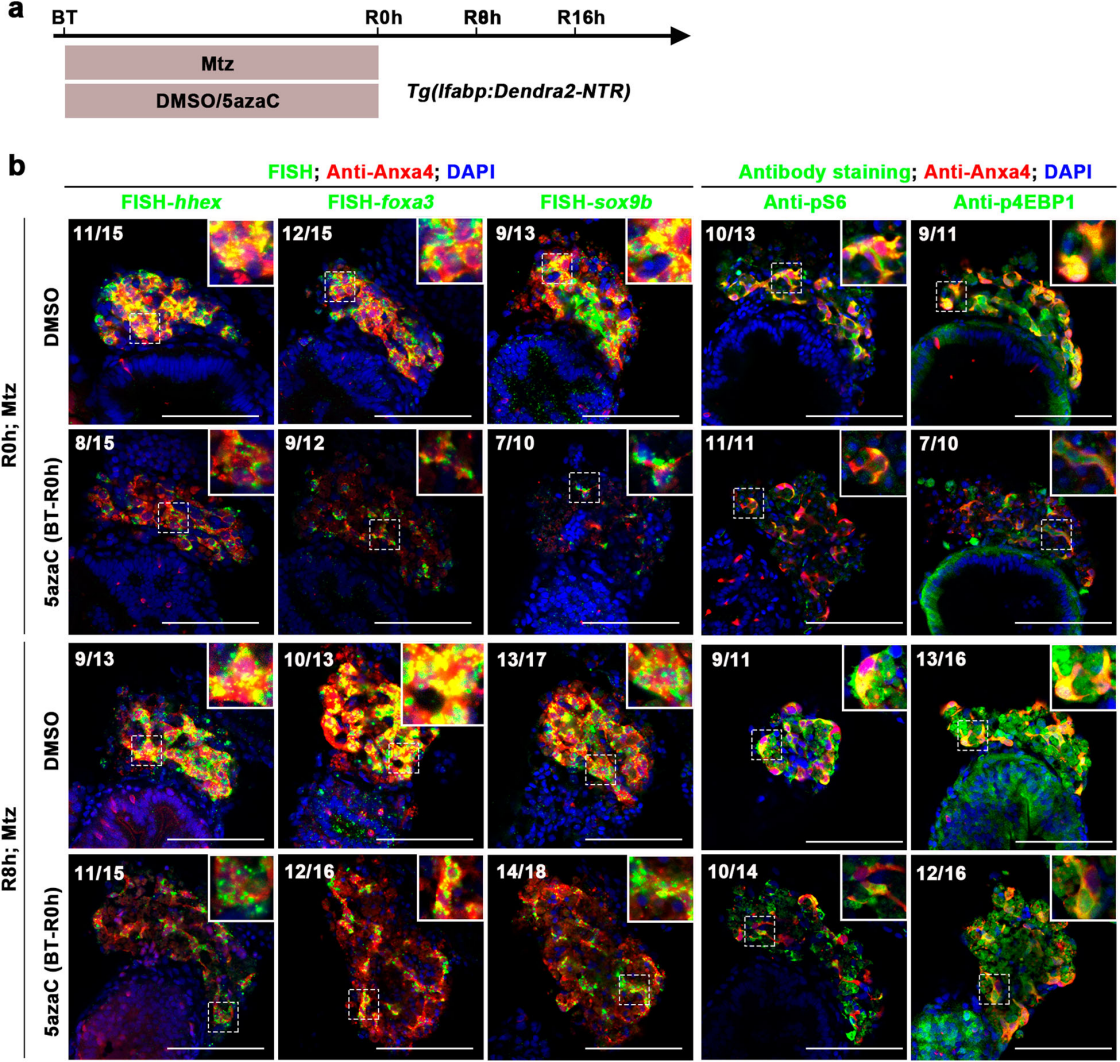
Early DNA methylation inhibition delays the dedifferentiation of BECs. **a** Experimental scheme illustrating the 5azaC and Mtz treatment and analysis at R0h and R8h. **b** FISH and antibody staining showing the expressions of hepatoblast markers *hhex, foxa3*, and *sox9b* and mTORC1 effectors pS6 and p4EBP1 in Anxa4+ cells (2D images) at R0h and R8h after early DNA methylation inhibition. Note that the expression levels of *hhex, foxa3, sox9b*, pS6, and p4EBP1 were downregulated in 5azaC-treated samples compared to controls. Numbers indicate the proportion of larvae exhibiting the expression shown. Scale bars: 100 µm. DAPI 4′, 6-diamidino-2-phenylindole, BT before treatment, FISH fluorescence in situ hybridization, R regeneration time after the withdrawal of Mtz.

To investigate the role of DNA methylation maintenance in the initiation stage of liver regeneration, we used the protocol of early 5azaC incubation during Mtz treatment from 5 dpf/BT to 6 dpf/R0h for 24 h (early inhibition of DNA methylation) (Fig. 2a and Supplementary Fig. 4a). Given that BECs firstly dedifferentiate into BPPCs after liver damage, with activations of endoderm and hepatoblast developmental markers *foxa3*, *hhex*, and *sox9b*^3,12^, we checked the effects of 5azaC on the BEC dedifferentiation at R0h. Fluorescent in situ hybridization (FISH) coupled with antibody staining^38^ showed that the transcriptional activations of *foxa3*, *hhex*, and *sox9b* were reduced at R0h and R8h in the 5azaC-treated groups compared to the controls (Fig. 2b), but became recovered at R16h (Supplementary Fig. 4b). These data indicate the delayed BEC dedifferentiation by the early inhibition of DNA methylation.

The mTORC1 signaling plays critical roles in activating BEC dedifferentiation after extensive hepatocyte damages^12^. Downregulation of DNMT1 has been reported to decrease the activation of mTORC1 signaling in kidney disease^39^. Therefore, we hypothesized that the activity of mTORC1 is regulated by DNA methylation. The phosphorylated ribosomal S6 protein (pS6) and phosphorylated 4E-binding protein 1 (p4EBP1), two well-established downstream effectors of mTORC1 signaling^40^, were analyzed. The expressions of pS6 and p4EBP1 were inhibited from R0h to R8h, but became recovered at R16h after early inhibition of DNA methylation (Fig. 2b and Supplementary Fig. 4b). These results suggest that early inhibition of DNA methylation delays the activation of mTORC1 signaling, indicating important roles of DNA methylation in the initiation of liver regeneration.

### DNA methylation maintenance at the *p53* locus promotes BEC dedifferentiation through derepressing mTORC1 signaling

**Fig. 3:**
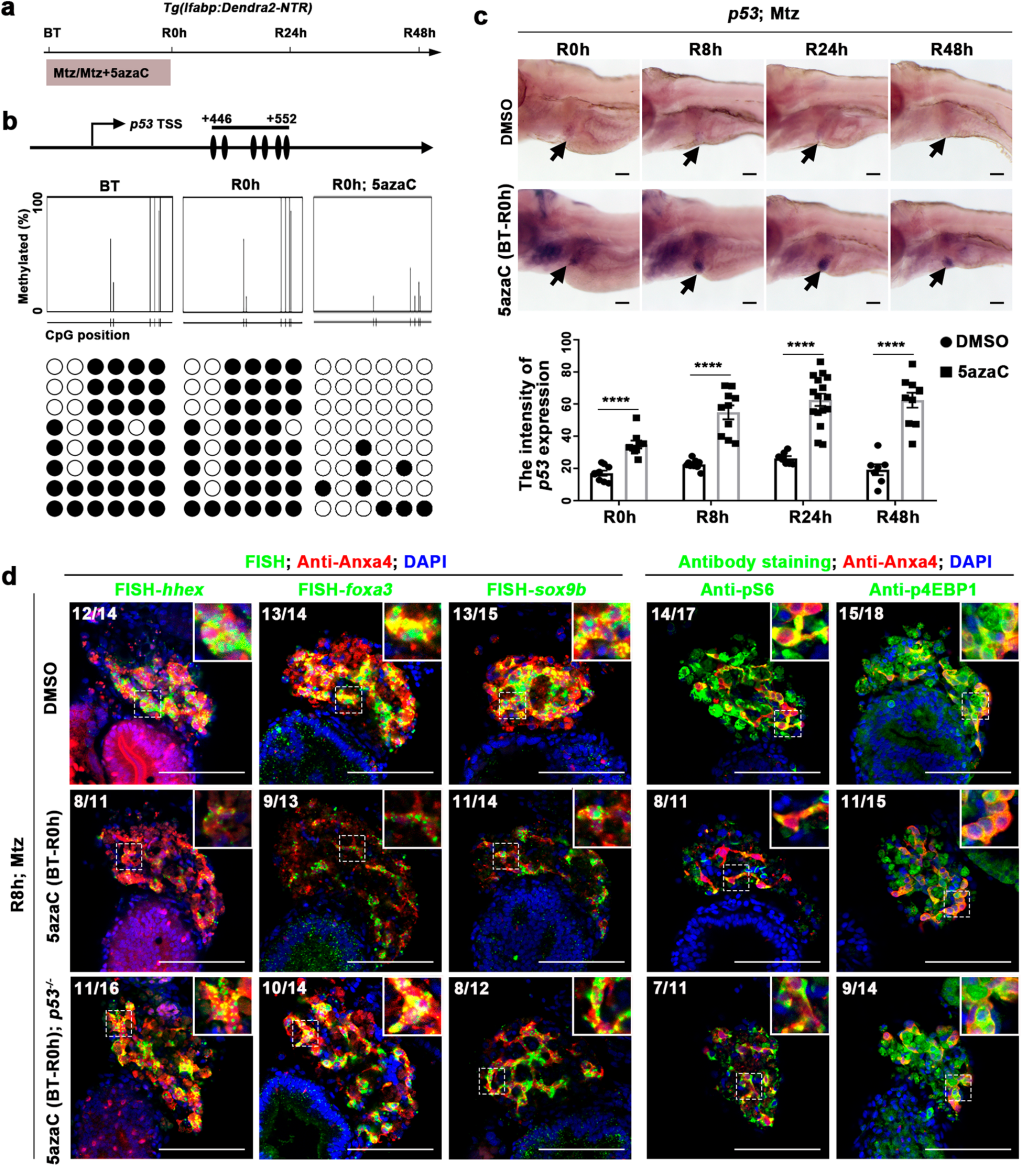
Early DNA methylation inhibition reduces BEC dedifferentiation through *p53* activation. **a** Experimental scheme illustrating the 5azaC and Mtz treatment and analysis from R0h to R48h. **b** Bisulfite sequencing analysis of DNA methylation at CpG islands near the promoter at the *p53* locus in DNA isolated from DMSO and 5azaC-treated livers at BT and R0h. **c** WISH images showing the expression of *p53* during liver regeneration after early DNA methylation inhibition. Quantification of the intensity of *p53* expression in liver regions from R0h to R48h. **d** Single-optical section images showing the expressions of hepatoblast markers *hhex*, *foxa3*, *sox9b*, and mTORC1 effectors pS6 and p4EBP1 at R8h in WT and *p53* mutant after early DNA methylation inhibition. Note that the expressions of hepatoblast markers and mTORC1 signaling were rescued in *p53* mutant compared with WT after early DNA methylation inhibition. Asterisks indicate statistical significance: ****P < 0.0001 using *t*-tests analysis when compared to control. Numbers indicate the proportion of larvae exhibiting the expression shown. Scale bars: 100 µm; error bars, ±SEM. DAPI 4′, 6-diamidino-2-phenylindole, BT before treatment, FISH fluorescent in situ hybridization, R regeneration time after the withdrawal of Mtz.

Dnmt1 has been reported to maintain pancreatic progenitor cell survival and regeneration by negatively regulating *p53*^30,41^. To investigate whether *p53* was aberrantly activated after early inhibition of DNA methylation, we examined DNA methylation at the *p53* locus at R0h with the analysis by MethPrimer^42^ and QUMA^43^ (Fig. 3a and b). In contrast to BT and R0h groups, 5azaC treatment led to significantly reduced level of DNA methylation at the *p53* locus (Fig. 3b). Thus, transcription of *p53* was upregulated from R0h to R48h (Fig. 3c; arrows). These results indicate that early inhibition of DNA methylation activates *p53* transcription during liver regeneration.

Aberrant activation of *p53* reduces the mTOR signaling in the setting of genotoxicities and stresses^44–46^. To explore whether DNA methylation regulates BEC dedifferentiation through *p53*, we analyzed hepatoblast markers and mTORC1 effectors in the *p53* mutant (Fig. 3d). The expressions of *hhex*, *foxa3*, *sox9b*, pS6, and p4EBP1 at R8h after early inhibition of DNA methylation were partially rescued in the *p53* mutant compared to the wild-type (WT) (Fig. 3d). The DNA methylation levels at the loci of *foxa3*, *hhex*, and *sox9b* were unaffected after 5azaC treatment, while the DNA methylation at the *p53* locus remained downregulated in the *p53* mutant after 5azaC treatment (Supplementary Fig. 5), indicating that DNA methylation at the *p53* locus is specifically regulated in the context of liver regeneration. These data indicate that DNA methylation at the *p53* locus derepresses the mTORC1 signaling and induces BEC dedifferentiation.

**Fig. 4:**
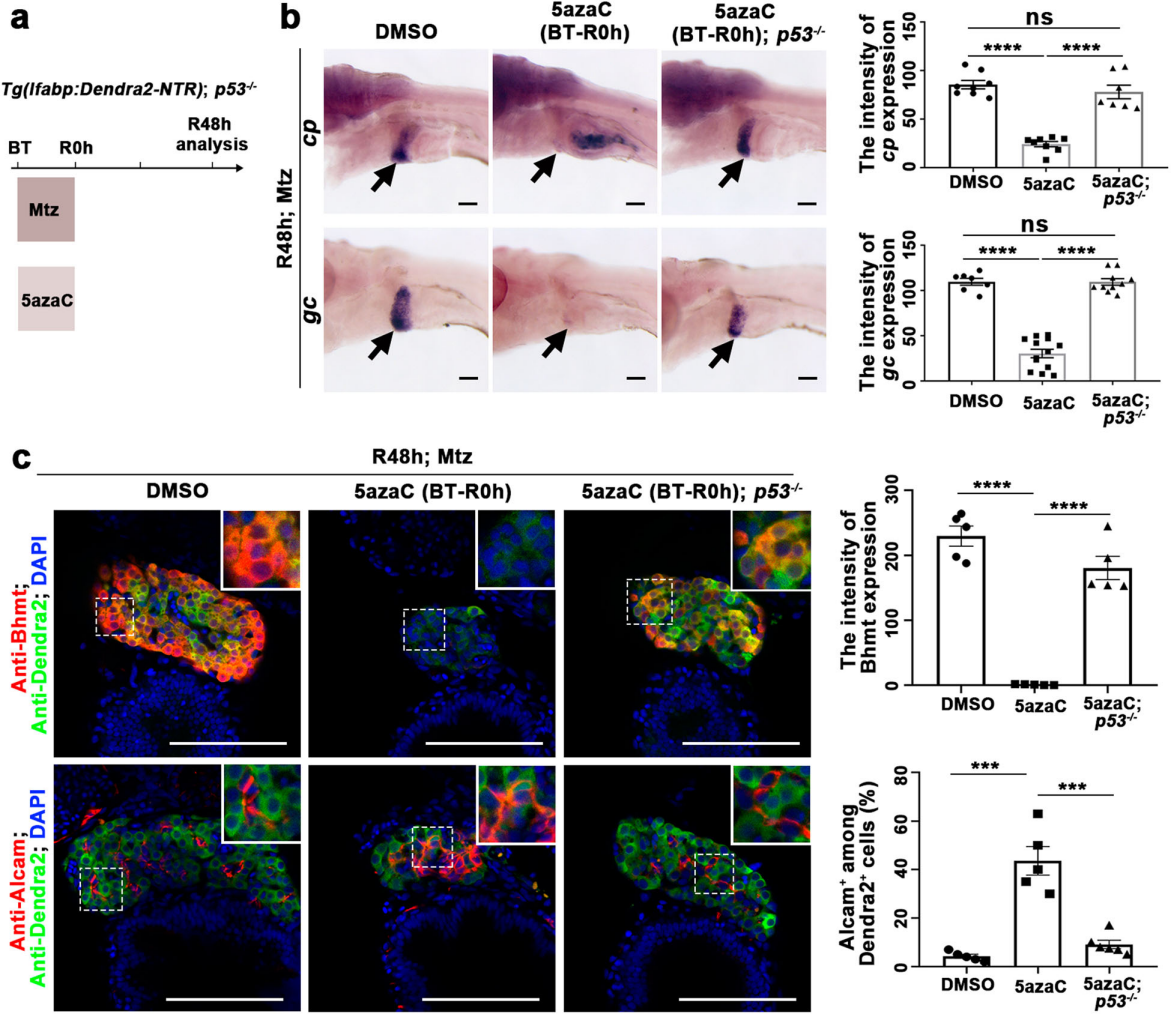
Loss of *p53* rescues liver regeneration defect after early DNA methylation inhibition. **a** Experimental scheme illustrating the 5azaC and Mtz treatment and analysis at R48h. **b** WISH images showing the expressions of hepatocyte markers *cp* and *gc* at R48h in WT and *p53* mutant after early DNA methylation inhibition. Note that the expressions of *cp* and *gc* were recovered in *p53* mutant after 5azaC treatment. Quantification of the intensity of *cp* and *gc* expression in liver regions at R48h. **c** Single-optical section images showing the expressions of hepatocyte marker Bhmt and BECs marker Alcam in WT and *p53* mutant after early DNA methylation inhibition and controls at R48h. Quantification of the intensity of Bhmt expression and the percentage of Alcam+ among Dendra2+ cells in liver regions at R48h. Note that the expression of Bhmt was recovered in *p53* mutant compared to WT after 5azaC treatment. Asterisks indicate statistical significance: ***P < 0.001; ****P < 0.0001 using *t*-tests analysis when compared to control. Scale bars: 100 µm; error bars, ±SEM. DAPI 4′, 6-diamidino-2-phenylindole, BT before treatment, ns no significant difference, R regeneration time after the withdrawal of Mtz.

To explore the effects of early inhibition of DNA methylation at the *p53* locus on liver regeneration, we checked the expressions of hepatocyte and BEC markers at R48h in the *p53* mutant. Mutation of *p53* rescued the loss of *cp* and *gc* expressions caused by 5azaC treatment (Fig. 4a and b). The expression of Bhmt and the ratio of Alcam^+^Dendra2^−^ mature BECs also recovered in the *p53* mutant (Fig. 4c). As a consequence, the reduced size of regenerating liver caused by the early incubation of 5azaC was partially rescued in the *p53* mutant (Supplementary Fig. 6a–c). The rescued hepatocyte regeneration by *p53* mutation after early 5azaC treatment did not affect the origin of nascent hepatocytes, which were also derived from BEC trans-differentiation (Supplementary Fig. 7). All these data indicate that DNA methylation at the *p53* locus regulates BEC-mediated liver regeneration.

### Maintenance of DNA methylation at the *p53* locus promotes BPPC redifferentiation at late stages of liver regeneration

**Fig. 5:**
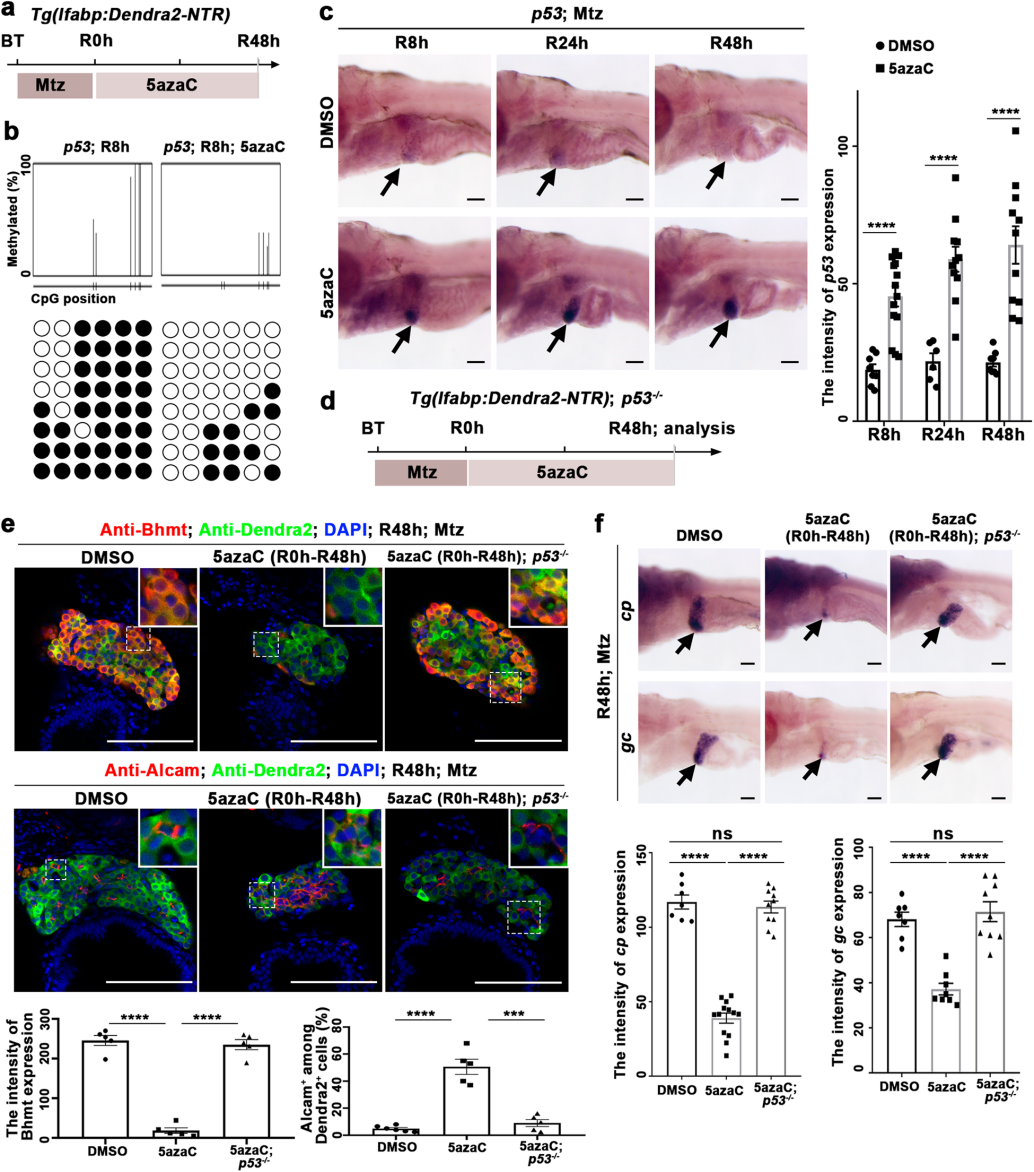
Late DNA methylation inhibition represses BPPC redifferentiation through *p53* activation. **a** Experimental scheme illustrating the 5azaC and Mtz treatment and analysis from R8h to R48h. **b** Bisulfite sequencing analysis of DNA methylation at the *p53* locus in DNA isolated from DMSO and 5azaC-treated livers. **c** WISH images showing the expression of *p53* from R8h to R48h during liver regeneration after late DNA methylation inhibition. Quantification of the intensity of *p53* expression in liver regions from R8h to R48h. **d** Experimental scheme illustrating the 5azaC and Mtz treatment and analysis at R48h in *p53* mutant and WT. **e** Single-optical section images showing the expressions of hepatocyte marker Bhmt and BECs marker Alcam at R48h. Quantification of the intensity of Bhmt expression and the percentage of Alcam+ among Dendra2+ cells in liver regions at R48h. **f** WISH images showing the expressions of *cp* and *gc* at R48h after 5azaC treatment in control and *p53* mutant after late DNA methylation inhibition. Quantification of the intensity of *cp* and *gc* expressions in liver regions at R48h. Note that the downregulation of *cp* and *gc* expressions was recovered in *p53* mutant after 5azaC treatment. Asterisks indicate statistical significance: ***P < 0.001; ****P < 0.0001 using *t*-tests analysis when compared to control. Scale bars: 100 µm; error bars, ±SEM. DAPI 4′,6-diamidino-2-phenylindole, BT before treatment, ns no significant difference, R regeneration time after the withdrawal of Mtz.

To evaluate whether DNA methylation is also involved in the late stages of liver regeneration, we used the protocol of late 5azaC treatment from R0h to R48h (late inhibition of DNA methylation) to bypass its earlier effects on BEC dedifferentiation (Fig. 5a). Late inhibition of DNA methylation reduced the sizes of regenerating liver at R24h and R48h (Supplementary Fig. 2c and 6b). Then, we analyzed whether these late effects on liver regeneration also involved by *p53*. Late 5azaC treatment reduced the level of DNA methylation on the *p53* locus at R8h (Fig. 5b), leading to elevated *p53* expression in the regenerating liver from R8h to R48h (Fig. 5c; arrows). To assess whether DNA methylation affects the BPPC redifferentiation through *p53*, we checked liver regeneration at R48h in the *p53* mutant (Fig. 5d and Supplementary Fig. 6a). The sizes of regenerating livers, the expression of hepatocyte makers *cp*, *gc*, and Bhmt, and the number of Alcam^+^Dendra2^-^ mature BECs reduced at R48h after late 5azaC treatment. By contrast, in the *p53* mutant, the sizes of regenerating livers and the redifferentiation from BPPCs to hepatocytes and BECs were partially rescued (Fig. 5e and f and Supplementary Fig. 6b and 6c). Similarly, all the rescued regenerating hepatocytes in the *p53* mutant were also derived from BEC trans-differentiation after late 5azaC treatment (Supplementary Fig. 7). These data indicate that loss of *p53* could rescue the defective liver regeneration caused by late inhibition of DNA methylation.

### DNA methylation at the *p53* locus activates BPPC redifferentiation through derepressing Bmp signaling

**Fig. 6:**
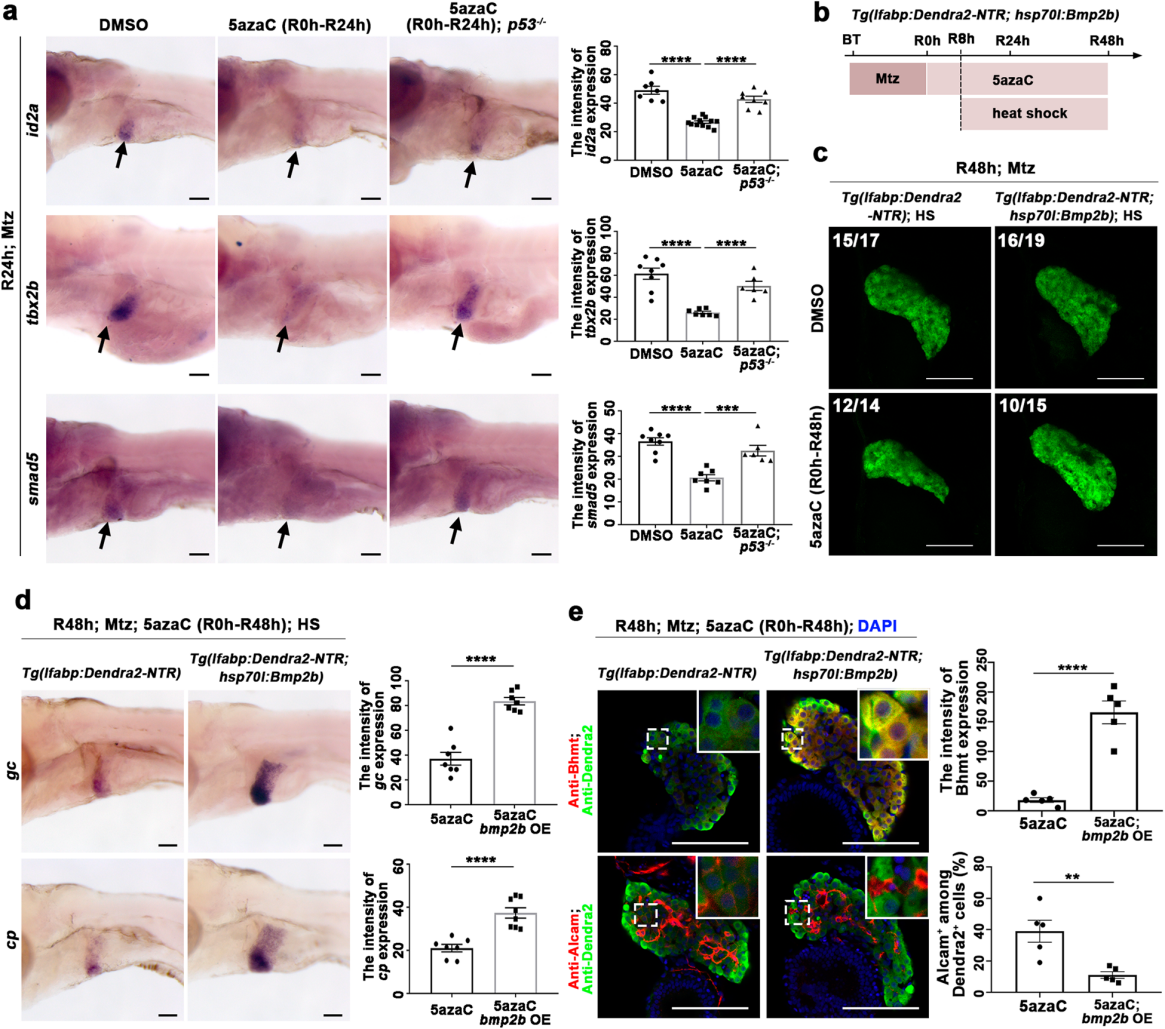
Late DNA methylation inhibition activates *p53* and in turn reduces BPPC redifferentiation through Bmp signaling. **a** WISH images showing the expressions of *id2a, tbx2b*, and *smad5* at R24h treated with DMSO or 5azaC in *p53* mutant and wild-type livers (arrows). Quantification of the intensity of *id2a, tbx2b*, and *smad5* expressions in liver regions at R24h. Note that the expressions of *id2a, tbx2b*, and *smad5* were recovered in *p53* mutant compared with control after 5azaC treatment. **b** Experimental scheme illustrating the 5azaC and Mtz treatment in transgenic line *Tg(lfabp:Dendra2-NTR; hsp70l:Bmp2b)* and heat shock from R8h to R48h. **c** Confocal images showing the regenerating livers at R48h after heat-shock and 5azaC treatment. **d** WISH images showing the expressions of *gc* and *cp* at R48h after heat-shock and 5azaC treatment. Quantification of the intensity of *gc* and *cp* expressions in liver regions at R48h. **e** Single-optical section images showing the expressions of hepatocyte marker Bhmt and BECs marker Alcam at R48h after heat-shock and 5azaC treatment. Quantification of the intensity of Bhmt expression and the percentage of Alcam+ among Dendra2+ cells in liver regions at R48h. Asterisks indicate statistical significance: **P < 0.01; ***P < 0.001; ****P < 0.0001 using *t*-tests analysis when compared to control. Numbers indicate the proportion of larvae exhibiting the expression shown. Scale bars: 100 µm; error bars, ±SEM. DAPI 4′,6-diamidino-2-phenylindole, BT before treatment, HS heat shock, OE overexpression, R regeneration time after the withdrawal of Mtz.

Bmp signaling governs BPPC redifferentiation through *tbx2b*, *id2a*, and *smad5* after extensive liver damages in zebrafish^20^. p53 regulates neural stem cell differentiation through BMP signaling^47^. So, we tested whether loss of DNA methylation at the *p53* locus could affect Bmp signaling during BPPC redifferentiation. The transcriptional activations of *id2a*, *tbx2b*, and *smad5*, three critical Bmp target genes^20^, were repressed in the regenerating livers at R24h after late 5azaC treatment, which were partially rescued in the *p53* mutant (Fig. 6a). To validate whether the effects of late 5azaC treatment on BPPC redifferentiation are mediated by the Bmp signaling, 5azaC was applied from R0h and *bmp2b* overexpression was induced by heat shock from R8h under the *Tg(hsp70l:Bmp2b)*^48^ transgenic background (Fig. 6b). Overexpression of *bmp2b* rescued the reduced size of regenerating liver in the 5azaC-treated group, but hardly accelerated normal liver regeneration in the control group (Fig. 6c). The expressions of mature hepatocyte and BEC markers were all rescued in the *bmp2b* overexpression groups after 5azaC treatment (Fig. 6d and e). In addition, the expressions of *foxa3*, *hhex*, and *sox9b*, as well as the mTORC1 effectors pS6 and p4EBP1, remained relatively normal at R24h after late 5azaC treatment, indicating that late inhibition of DNA methylation is ineffective to mTORC1 activation and BEC dedifferentiation (Supplementary Fig. 8). All these results indicated that DNA methylation at the *p53* locus promotes BPPC redifferentiation through Bmp signaling.

### The *dnmt1* mutant exhibits aberrant *p53* activation and impaired BEC dedifferentiation and BPPC redifferentiation

**Fig. 7:**
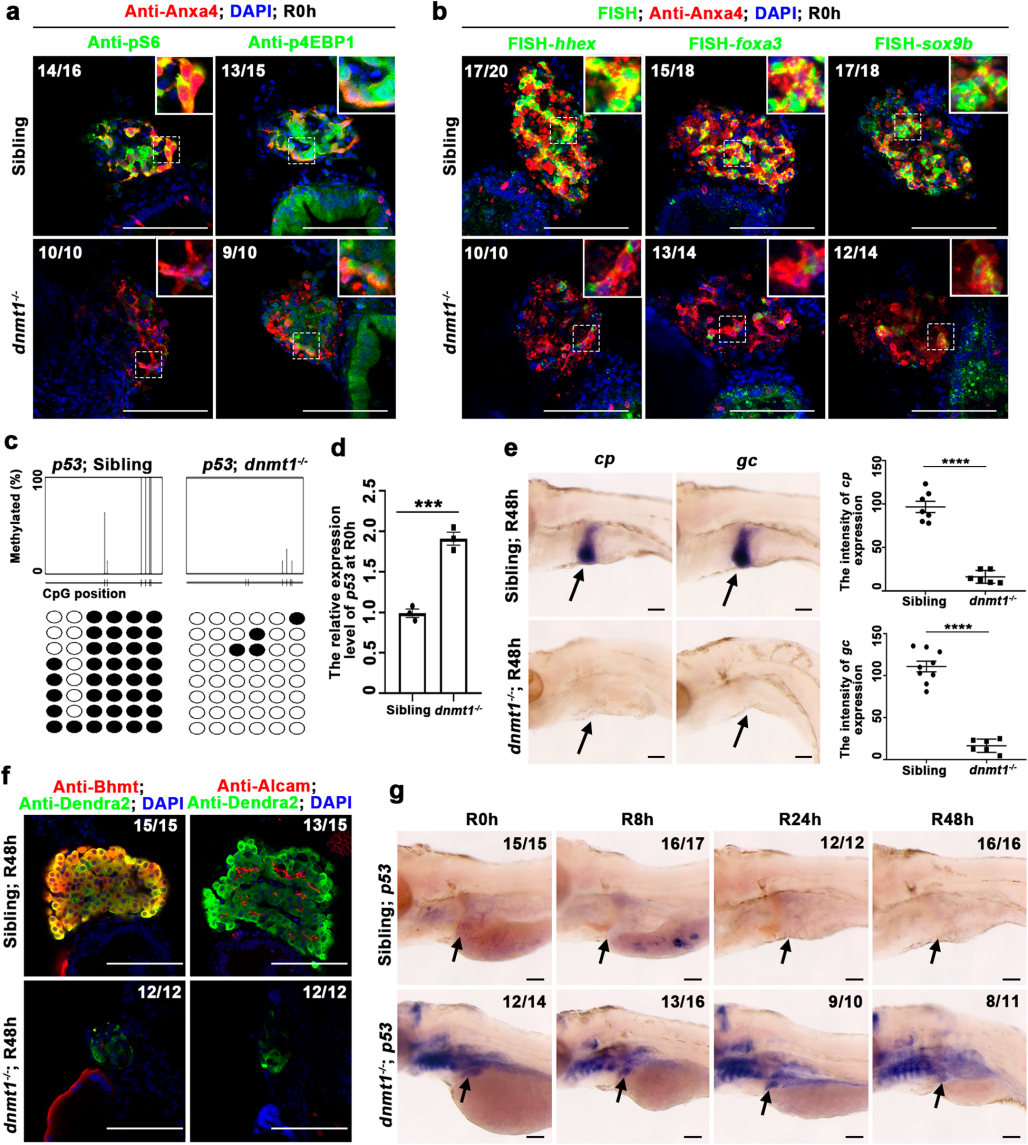
The *dnmt1* mutant blocks liver regeneration. **a** Single-optical section images showing the expression of pS6, p4EBP1, and Anxa4 in regenerating livers at R0h in *dnmt1* mutant and sibling. Note that the mTORC1 signaling effectors pS6 and p4EBP1 are downregulated in *dnmt1* mutant compared with the sibling. **b** Single-optical section images showing the FISH and antibody staining images of the expressions of *hhex*, *sox9b*, *foxa3*, and Anxa4 in regenerating livers at R0h in *dnmt1* mutant and sibling. **c** Bisulfite sequencing analysis of DNA methylation at the *p53* locus in DNA isolated from livers of *dnmt1* sibling and mutant at R0h. **d** qPCR data showing the relative expression level of *p53* in livers of *dnmt1* mutant and sibling at R0h. **e** WISH images showing the expressions of *cp* and *gc* at R48h in *dnmt1* mutant and sibling livers (arrows). Quantification of the intensity of *gc* and *cp* expressions in liver regions at R48h. **f** Single-optical section images showing the expressions of hepatocyte marker Bhmt, BECs marker Alcam, and Dendra2 in regenerating livers at R48h in *dnmt1* mutant and sibling. **g** WISH images showing the expression of *p53* at R0h, R8h, R24h, and R48h in *dnmt1* mutant and sibling livers (arrows). Asterisks indicate statistical significance: ***P < 0.001; ****P < 0.0001 using *t*-tests analysis when compared to control. Numbers indicate the proportion of larvae exhibiting the expression shown. Scale bars: 100 µm; error bars, ±SEM. DAPI 4’,6-diamidino-2-phenylindole, FISH fluorescent in situ hybridization, R regeneration time after the withdrawal of Mtz.

To further substantiate the roles of DNA methylation in liver regeneration, we checked the liver regeneration in the *dnmt1*^*s872*^ mutant^30^. The *dnmt1* mutant displays liver degeneration^30^ from 5 dpf (Supplementary Fig. 9a). To exclude the side effect of liver degeneration, we ablated the hepatocytes with Mtz from 4 dpf for 24 h, then checked the liver regeneration. Most of the *dnmt1* mutants survived to R48h, but liver regeneration was defective (Supplementary Fig. 9a and 9b). The expression level of 5meC in Tomato-positive BPPCs was downregulated in the *dnmt1* mutant at R0h (Supplementary Fig. 9c). To exclude the abnormal liver development at late stages of liver regeneration, we injected *dnmt1* mRNA into the *dnmt1* mutant, then examined liver development and regeneration from 4 dpf to R48h (Supplementary Fig. 10a). Because the injected mRNA could maintain in the embryo only for 3–4 days, the injected *dnmt1* mRNA partially rescued the defective liver development at 4 dpf, but failed to rescue later liver regeneration (Supplementary Fig. 10). The dedifferentiation was arrested in the *dnmt1* mutant as assessed by the expression of mTORC1 effectors and hepatoblast markers at R0h (Fig. 7a and b). The DNA methylation level at the *p53* locus was reduced, and the transcriptional level of *p53* was upregulated in the *dnmt1* mutant at R0h (Fig. 7c and d). These data indicate that *dnmt1* is important for BEC dedifferentation.

To further access the roles of *dnmt1* in the late stages of liver regeneration, we analyzed BPPC redifferentiation in the *dnmt1* mutant. The expressions of hepatocyte markers including *cp*, *gc*, and Bhmt, as well as the BEC marker Alcam, were undetectable in the *dnmt1* mutant at R48h (Fig. 7e and f). The expressions of *p53* itself, as well as its target genes *p21* and *mdm2*, were upregulated in the liver of *dnmt1* mutant from R0h to R48h (Fig. 7g and Supplementary Fig. 11). Taken together, these results suggest that *dnmt1* is important for both BEC dedifferentiation and BPPC redifferentiation through the inactivation of *p53*.

### Aberrant activation of *p53* impairs the BEC-mediated liver regeneration

**Fig. 8:**
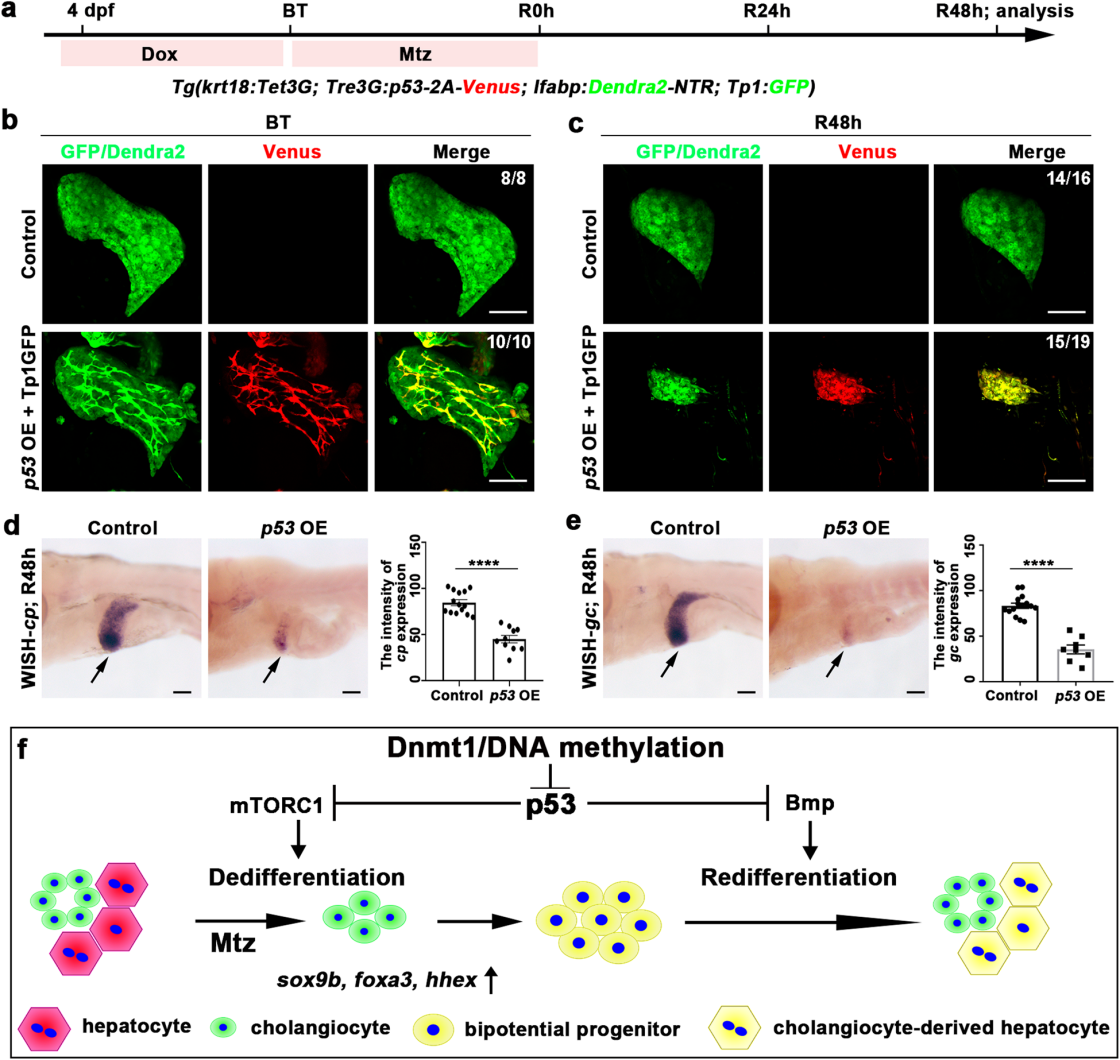
BEC-specific *p53* activation reduces liver regeneration. **a** Experimental scheme illustrating the time point of Dox and Mtz treatment and analysis at R48h in the transgenic line *Tg(krt18:Tet3G; Tre3G:p53-2A-Venus; lfabp:Dendra2-NTR; Tp1:GFP)*. **b** Confocal images showing the expressions of Venus and Dendra2 in regeneration livers at BT in control and *p53* overexpression groups. Note that *p53* expresses specifically in GFP strong BECs after Dox treatment. **c** Confocal images showing the expressions of Venus and Dendra2 in regeneration livers at R48h in control and *p53* overexpression groups. Note that *p53* overexpression reduces liver regeneration at R48h. **d** WISH images showing the expressions of *cp* at R48h after *p53* overexpression. Quantification of the intensity of *cp* expressions in liver regions at R48h. **e** WISH images showing the expressions of *gc* at R48h after *p53* overexpression. Quantification of the intensity of *gc* expressions in liver regions at R48h. **f** A model summarizing our finding that the maintenance of DNA methylation at the *p53* locus by Dnmt1 regulates BECs-mediated liver regeneration through mTORC1 and Bmp signaling. Asterisks indicate statistical significance: ****P < 0.0001 using *t*-tests analysis when compared to control. Numbers indicate the proportion of larvae exhibiting the expression shown. Scale bars: 100 µm; error bars, ±SEM. BT before treatment, Dox Doxycycline, dpf days post-fertilization, OE overexpression, R regeneration time after the withdrawal of Mtz, WISH whole-mount in situ hybridization.

To determine the roles of *p53* in liver regeneration, we used the Tet-on system^49^ to specifically overexpress *p53* in BECs under the *Tg(Krt18:Tet3G; Tre3G:p53-2A-Venus; lfabp:Dendra2-NTR; Tp1:GFP)* transgenic background (Fig. 8a). The Doxycycline (Dox) treatment from 4 dpf to BT induced overexpression of p53 in the *Tp1:GFP*^+^ BECs at BT, which led to reduced liver regeneration at R48h (Fig. 8b–e). General overexpression of *p53* was achieved by heat shock under the *Tg(hsp70l:p53-2A-Venus)* transgenic background (Supplementary Fig. 12a and 12b), which led to defective liver regeneration (Supplementary Fig. 12c). The *p53* overexpression from BT to R0h reduced the mTORC1 signaling and the expression of hepatoblast markers at R0h (Supplementary Fig. 13a–c), indicating impaired BEC dedifferentiation and BPPC formation. Furthermore, overexpression of *p53* from R0h to R24h repressed Bmp signaling at R24h (Supplementary Fig. 13d and 13e). These data demonstrate that aberrant *p53* activation impairs early and late stages of liver regeneration through mTORC1 and Bmp signaling, respectively.

### The maintenance of DNA methylation is essential for ductular reaction in mice

The roles of DNA methylation in BEC dedifferentiation in zebrafish prompted us to check its effects in mice. We made a pilot study using a choline-deficient, ethionine-supplemented (CDE) diet to investigate the ductular reaction in mice, in which hepatic progenitor cells are formed from BECs^50^. Mice fed with CDE diet for 10 days were subjected to 5azaC or PBS injection from day 3 to day 9 (Supplementary Fig. 14a). Liver damage was comparable between vehicle and 5azaC-injected mice as indicated by Serum alanine aminotransferase (ALT), aspartate aminotransferase (AST), and bilirubin (Supplementary Fig. 14b). The level of DNA methylation was high in BECs in the chow control, and maintained in the KRT19^+^ ductular cells after liver injury (Supplementary Fig. 14c). The 5azaC treatment significantly reduced the level of DNA methylation in the ductular cells, but not in other hepatic cells (Supplementary Fig. 14c). To verify the effects of DNA methylation on the formation of progenitors, we examined the expression patterns of SOX9, a well-known hepatic progenitor cell marker^50^ and KRT19. The expression areas of SOX9 and KRT19 were obviously reduced in the 5azaC-injected mice (Supplementary Fig. 14d), indicating the importance of DNA methylation in ductular reaction. Altogether, these mouse data indicate that the maintenance of DNA methylation regulates the formation of progenitor cells as in zebrafish.

## Discussion

Zebrafish as an animal model is widely used to explore the cellular and molecular mechanisms underlying organ development and tissue regeneration^3,51–57^. For the zebrafish, extensive hepatocyte damages and mouse extensive liver injury models allow the evaluation of cellular and molecular mechanisms during liver regeneration in end-stage liver diseases. Uncovering the detailed mechanisms underlying the BEC dedifferentiation and redifferentiation are crucial to promoting BECs-mediated liver regeneration. Here, we describe that DNA methylation inhibition from BT-R0h and *dnmt1* mutants reduces the dedifferentiation of BECs with the activation of *p53* after liver injury. Moreover, DNA methylation inhibition from R0h to R48h reduces the BPPC redifferentiation. Hypermethylation of *p53* regulates the dedifferentiation of BECs by mTORC1 signaling and induces BPPC redifferentiation through Bmp signaling. Loss of *p53* rescues BPPCs activation and redifferentiation in liver regeneration after inhibition of DNA methylation (Fig. 8f). Furthermore, we show that DNA methylation may play a role in mouse hepatic progenitor cell activation.

Epigenetic regulation has an essential role in the progress of liver regeneration. Histone deacetylation enzyme (Hdac1) regulates differentiation of BPPCs after extensive hepatocyte damages in zebrafish^23^. The DNA methylation factor DNMT1 maintains the liver homeostasis and regeneration after partial hepatectomy (PH) in mice^34^. Another DNA methylation regulator, Uhrf1, mediates liver regeneration after PH in zebrafish^58^ and mice^35^. Although DNA methylation is vital for liver development^30,58,59^, we found the dedifferentiation of BECs, the redifferentiation of BPPCs were blocked in *dnmt1* mutant and DNA methylation inhibition. Indeed, knockdown of *Dnmt1* in cultured hepatic progenitor cells leads to sphere formation defect^34^. Although the other hepatic cells may also express high level of DNA methylation and play roles in liver regeneration, we found Dnmt1 is specifically upregulated in BPPCs after liver injury. These data indicate Dnmt1 regulates liver regeneration in a cell-autonomous manner. The DNA methylation levels are slightly downregulated from R0h to R24h in BPPCs, which validates that the DNA hypomethylation of some other genes may also play essential roles during liver regeneration^24^, suggestings that different genes with distinct DNA methylation levels on their promoters contribute to liver regeneration together.

The dedifferentiation of BECs and the activation of mTORC1 signaling are reduced after early DNA methylation inhibition, loss of *p53* partially rescues the expressions of mTORC1 signaling and hepatoblast markers in BPPCs at R8h. Based on the crucial roles of mTORC1 signaling in the early stage of liver regeneration^12,14^, other compensation pathways may involve in regulating the mTORC1 signaling after DNA hypomethylation. Indeed, epigenetic compensation has been reported to promote liver regeneration after DNA methylation inhibition^35^. Some stem genes and regeneration pathways may be activated to induce liver regeneration after DNA hypomethylation^24^. The factors, such as *sesn1/2*, *cab39l*, and *ddit3/4*, may play roles in inhibiting the activation of mTORC1 signaling^39,46,60^. *P53* and *Ddit4* synergetically license differentiated cells to the cell cycle by suppressing mTORC1 signaling in tissue regeneration^60^. It may be the reason why the only loss of one gene, such as *p53*, partially rescues the depression of mTORC1 signaling after inhibition of DNA methylation. Accordingly, *p53* activation negatively regulates zebrafish heart regeneration^61^. Our results indicate the mechanisms by which Dnmt1 mediates BPPCs activation, in part, through the regulation of *p53* signaling. Whether other factors that Dnmt1 transcriptionally represses are involved in this progress needs further exploration.

Dnmt1 controls the expression of *p53* both in organ development and regeneration^30,41^. Inhibition of DNA methylation from BT to R0h and R0h to R48h activates the expression of *p53*. The roles of *p53* in cell apoptosis have been well-known^34,41^, but whether *p53* affects biliary-derived liver regeneration is less addressed. The liver stem cells develop into hepatocellular carcinoma in *p53*-deficient mice^62^, and our results show normal liver regeneration in zebrafish *p53* mutant. This discrepancy may be caused by the short time of liver regeneration (about 48 h) in zebrafish liver regeneration. BEC-specific and whole-body overexpression of *p53* reduce liver regeneration, indicating *p53* regulates biliary-derived liver regeneration in a cell-autonomous manner. The sizes of regenerating liver and the expressions of hepatocyte markers decreased after strong genetic overexpression of *p53*. Indeed, *p53* overexpression during liver injury reduces the activity of mTORC1 in BPPCs, and *p53* overexpression in BPPC redifferentiation reduces Bmp signaling, suggesting *p53* regulation is stage-dependent. *p53* has been reported to regulate BMP signaling^47,63^. However, how p53 represses mTORC1 and Bmp signaling during liver regeneration needs to be further explored.

Above all, this study explored the roles of DNA methylation in BECs-mediated liver regeneration. DNA methylation at the *p53* locus represses *p53* transcription and in turn derepresses mTORC1 signaling to activate BEC dedifferentiation. After BEC dedifferentiation and BPPCs formation, DNA methylation at the *p53* locus maintains in BPPCs to continue blocking *p53* transcription, which derepresses Bmp signaling to induce BPPC redifferentiation. This study indicates the roles of DNA methylation and *p53* as potential targets for therapeutics after severe acute liver injuries.

## Methods

### The agency or committee that granted approval

All animals are housed in a temperature- and light-controlled facilities and are maintained in accordance with the Guide for Care and Use of Laboratory Animals and the Animal Welfare Act. Experiments were performed with the approval of the Institutional Animal Care and Use Committee (IACUC) at Southwest University.

### Transgenic fish lines

Zebrafish *p53* full-length coding sequence was cloned from 3 days post-fertilization (dpf) cDNA with primers *p53* fw: 5′-cctgatcgataccgtcgacatggcgcaaaacgacagccaag-3′ and *p53* rv: 5′-gtagctccgcttccgctagcatcagagtcgcttcttccttc-3′. *hsp70l:p53-2a-venus* construct was generated by replacing the *Rps6kb1b-2A-HA-Rps6* (unpublished) with *p53* by the NovoRec PCR step directional cloning kit (Novoprotein, China). *Tre3G:p53-2A-Venus* construct was generated from *Tre3G:kras* (unpublished) by inserting the *p53-2A-Venus* digested with *BamH*I (NEB) and *Not*I (NEB) enzymes. *Krt18:tet3G* construct was generated from *lfabp:tet3G* plasmid (unpublished) by replacing the *lfabp* promoter with *krt18* promoter with *BsmB*I (NEB). *Krt18:tet3G, tre3G:p53-2A-Venus* or h*sp70l:p53-2a-venus* flanked by the *I-Sce*I restriction sites were co-injected with *I-Sce*I (NEB) into the cell of one-cell stage embryos of the *Tg(lfabp:Dendra2-NTR)*^*cq1*^ background. Candidate founder embryos were selected based on specific expression of CFP in the eyes were raised to adulthood. These zebrafish were screened for germ-line transmission and chose the best representative transgenic line for the following experiments. The fish strains and detailed information are listed in Supplementary Table 1.

### *Dnmt1* mRNA microinjection

Zebrafish *dnmt1* full-length coding sequence was cloned from 3 dpf cDNA. The *dnmt1* PCR product was subcloned into the *pCS2(+)* vector by the NovoRec PCR step directional cloning kit (Novoprotein, China) for RNA preparation. Sense mRNA was synthesized from a linearized plasmid template using the mMESSAGE mMACHINE Kit (Ambion). 150 pg *dnmt1* mRNA injected into the cell of the one-cell stage embryos. The primers used in this study are listed in Supplementary Table 2.

### 5-Azacytidine and metronidazole treatment

The *Tg(lfabp:Dendra2-NTR)*^*cq1*^ transgenic larvae were incubated with 10 mM Mtz (Sigma–Aldrich) in 0.2% dimethylsulfoxide (DMSO) from 5 days post-fertilization (dpf) to 6 dpf for 24 h. The *dnmt1* mutants were treated with Mtz from 4 dpf to 5 dpf for 24 h. Then, larvae were washed and recovered in egg water, marking the regeneration 0 h (R0h). For 5-Azacytidine (5azaC) (Selleck) treatment, larvae were treated with 100 µM 5azaC in 0.2% DMSO from 5 dpf to R0h or R0h to R48h. Then, the larvae were washed three times and recovered in egg water.

### Temporal control of CreER activities

*Tg(krt18:CreER; βactin:loxP-DsRed-loxP-GFP)* and *Tg(krt18:CreER; lfabp:loxP-STOP-loxP-DsRed; lfabp:Dendra2-NTR)* larvae were treated with 5 µM 4-hydroxytamoxifen (4OHT; Sigma) in egg water at 28.5 °C for 24 h from 4 to 5 dpf, followed by three washes with fresh egg water.

### Doxycycline induction

*Tg(krt18: tet3G; Tre3G: p53-2A-Venus; lfabp:Dendra2-NTR; Tp1:GFP)* larvae were incubated with 20 µg/ml doxycycline (Sangon Biotech, China) in egg water at 28.5 °C for 24 hours from 4 dpf to BT (5 dpf), followed by three washes with fresh egg water.

### Heat-shock treatment

*Tg(hsp70l:p53-2a-venus)* and *Tg(hsp70l:Bmp2b)* larvae were heat-shocked at 38.5 °C for 35–60 min in water bath and then incubated at 28.5 °C for gradual recovery.

### Whole-mount antibody staining

For whole-mount antibody staining, larvae were removed the skin with the Tweezers, washed several times with PT (PBS + 1% Triton X-100) and incubated with primary antibodies below: Anxa4 (1:1000; ab71286, Abcam, Cambridge, MA), 5-methyl-cytosine (1:100; ab10805, Abcam, Cambridge, MA), Alcam (1:50; zn5, ZIRC, Eugene, OR), Bhmt (1:500, a kind gift from Jinrong Peng, Zhejiang University, China), Dendra2 (1:1000; AB821, Evrogen, Moscow, Russia), pS6 (1:500; 2215, Cell Signaling, MA, USA), p4EBP1 (1:500; 2855, Cell Signaling, MA, USA), Dnmt1 (1:200, sc-20701, Santa Cruz Biotechnology, Santa Cruz, CA, a kind gift from Jingwei Xiong, Peking University, China), GFP (1:1000, ab6658, Abcam, Cambridge, MA), DsRed (1:500, sc-101526, Santa Cruz Biotechnology, Santa Cruz, CA) and Tomato (1:1000; orb182397, Biorbyt, TX, USA), After primary antibody incubation, larvae were washed several times with PT and incubated with secondary antibodies conjugated to Alexa Fluor 488/568/633 (1:1000; Invitrogen, Grand Island, NY). The primary and secondary antibodies were diluted in the blocking solution (PBS + 4% BSA + 1% Triton X-100) and incubated at 4 °C overnight and washed with PT for five times.

### Mouse studies

Four-week-old female C57BL/6-WT mice were housed under pathogen-free conditions under 12 h light-dark cycles. All experiments were performed following the guidelines of the IACUC at Southwest University. The mice were given Choline Deficient Chow (XieTong Biotech, Nanjing, China) and drinking water containing 0.15% (wt/vol) DL-ethionine (TCI). After 3 days on the CDE diet, animals received daily intraperitoneal injections of the 5azaC (Selleck) dissolved in the PBS at a dose of 2.5 µg/g. After 7 days of injections, the mice were sacrificed and liver tissue was collected and fixed in 4% PFA for analysis.

For immunohistochemical staining, the mouse liver tissue was embedded in 4% low melting point agarose; then the tissue was sliced into 40 µm sections. The sections underwent antigen retrieval in sodium citrate buffer (pH 6.0) or Tris-EDTA buffer (pH 8.5) in a Dry Bath Incubator for 10 minutes before blocking and permeabilization. The sections were permeabilized using 1% Triton X-100/PBS for 2 h at room temperature, followed by blocking with PBTN for 1 h at room temperature. Then the sections were incubated with the KRT19 primary antibody (1:500; ab133496; Abcam, Cambridge, MA), SOX9 primary antibody (1:300; A5080, Bimake), and 5meC primary antibody (1:100; ab10805, Abcam, Cambridge, MA) overnight at 4 °C. (For immunohistochemical staining of 5meC primary antibody, sections were incubated with 2 N HCl overnight at room temperature and then neutralized in 100 mM Tris-HCl (pH 8.5) for 1 h).

### Whole-mount in situ hybridization

For Whole-mount in situ hybridization, cDNA from the whole embryos at 3 dpf, R0h, and R48h were used as a template to amplify the genes by PCR. Digoxygenin (DIG) labeled antisense probes were synthesized from PCR products using the T7 or SP6 RNA polymerase (Roche). Larvae at the desired stages were fixed with 4% paraformaldehyde (PFA) in PBS at 4 °C overnight, then dehydrated by methanol and stored at −20 °C overnight. The larvae were then rehydrated successively in 75, 50, 25, and 0% methanol in PBT (0.1% Tween-20 in PBS), then prehybridized the larvae in HYB (50% formamide, 5 × SSC, 0.1% Tween-20, 5 mg/ml torula yeast RNA, 50 mg/ml heparin) at 68.5 °C for 3–5 h. The HYB was replaced with antisense probes diluted in HYB and hybridized at 68.5 °C overnight. The larvae were gradually washed at 68.5 °C with 100, 75, 50, and 25% HYB in 2 × SSCT, and finally in 0.2 × SSCT. Then the larvae were progressively replaced with 25, 50, 75, and 100% MABT (150 mM maleic acid, 100 mM NaCl, 0.1% Tween-20, pH 7.5) in 0.2 × SSCT, and blocked in 2% Block Reagent (Roche) at room temperature for 2 h. Larvae were then incubated with anti-Dig-AP antibody (Roche) diluted at 1/2000 with blocking buffer at 4 °C overnight. Larvae were rinsed with MABT for eight times, then staining with NBT/BCIP solution (Roche). The WISH images were captured using a SteREO DiscoveryV20 microscope equipped with AxioVision Rel 4.8.2 software (Carl Zeiss, Jena, Germany). The primers are listed in Supplementary Table 2.

### Fluorescence in situ hybridization coupled with antibody staining assays

For fluorescence in situ hybridization couple antibody staining, larvae were fixed with 4% PFA in PBS at 4 °C overnight, then incubated in 100% methanol at −20 °C for 24 h. Larvae were rehydrated into PBT and the larvae skins were manually peeled off with tweezers. Next, the larvae were washed five times with PBST (1% Triton X-100 in PBS) for 1 h, then prehybridized the larvae in HYB at 65 °C for 3–5 h. The HYB was replaced with new HYB containing Digoxigenin-labeled antisense probes and hybridized at 65 °C overnight. The following day, the larvae were washed into 2 × SSCT and 0.2 × SSCT at 65 °C. Then the larvae were replaced with MABT, and blocked for 2–3 h at room temperature with 2% Block Reagent (Roche) in MABT. Larvae were incubated in Anti-Dig POD antibody solution (Roche) diluted at 1/1000 with blocking buffer at 4 °C overnight. The next day larvae were progressively washed with MABT for eight times, with PBST for four times, with PBS twice, and then incubated with TSA Plus Fluorescein Solution (Perkin Elmer) overnight at room temperature with gentle shake. The following day, the larvae were washed with PBST for five times and then performed the antibody staining assays. The primers are listed in Supplementary Table 2.

### Quantitative-PCR

Total RNA was extracted from 100 to 200 dissected livers using the TRIzol reagent (Roche). cDNAs were synthesized from total RNA by using the Reverse Transcription Kit (Promega) following the manufacturer’s instructions. Quantitative real-time PCRs (qPCRs) were performed using the Fast Start Universal SYBR Green Master (Roche), and normalized by *eef1a1* in triplicate. The primers used in this study are listed in Supplementary Table 2.

### Genomic DNA isolation, bisulfite conversion, and sequencing

Liver genomic DNA was extracted from 100 to 150 dissected livers using lysis buffer containing 100 µg/ml Proteinase K at 55 °C for 4 h. Then, genomic DNA was purified with phenol: chloroform: isoamyl alcohol (25:24:1), and ethanol. The purified DNA was performed bisulfite conversion following the manufacturer’s instructions with the EpiTect Bisulfite Kit (Qiagen). The promoter sequence was amplified from Bisulfite-converted DNA using primers designed by MethPrimer. PCR products were purified and cloned into the *pGEM-T* easy vector (Promega). Eight colonies were sequenced for each sample using M13F sequencing primer. QUMA was used to analyze the sequencing results. The primers used in this study are listed in Supplementary Table 2

### Image acquisition and processing

The zebrafish larvae were imaged using a SteREO DiscoveryV20 microscope equipped with AxioVision Rel 4.8.2 software (Carl Zeiss). Antibody-stained and live larvae were imaged using ZEN 2010 software equipped on an LSM880 confocal microscope (Carl Zeiss). All figures, labels, arrows, scale bars, and outlines were drawn using Adobe Photoshop software.

### Quantification and statistical analysis

Automated quantification of the intensity of whole-mount in situ hybridization was performed using ImageJ 1.47 software (National Institute of Health). The fluorescence intensity and the area of liver sizes were quantified with ZEN 2010 (Zeiss). Unpaired two-tailed Student’s *t*-test was used for statistical analysis by GraphPad Prism 7; *P* < 0.05 was considered statistically significant. Quantitative data were shown as means ± SEM.

### Reporting summary

Further information on research design is available in the Nature Research Reporting Summary linked to this article.

## Supplementary information

Supplemental information

REPORTING SUMMARY

## Data Availability

The data that support the findings of this study are available from the corresponding author upon reasonable request.
