# Supplementary material for: DNA methylation maintenance at the p53 locus initiates biliary-mediated liver regeneration
Source: NPJ Regen Med. 2022 Mar 29;7:21. doi: 10.1038/s41536-022-00217-8 (PMC8964678; doi:10.1038/s41536-022-00217-8)
Supplement: Supplementary file 1 — Supplemental information [file 41536_2022_217_MOESM1_ESM.pdf]

Supplementary Information

Supplementary Figures and Legends

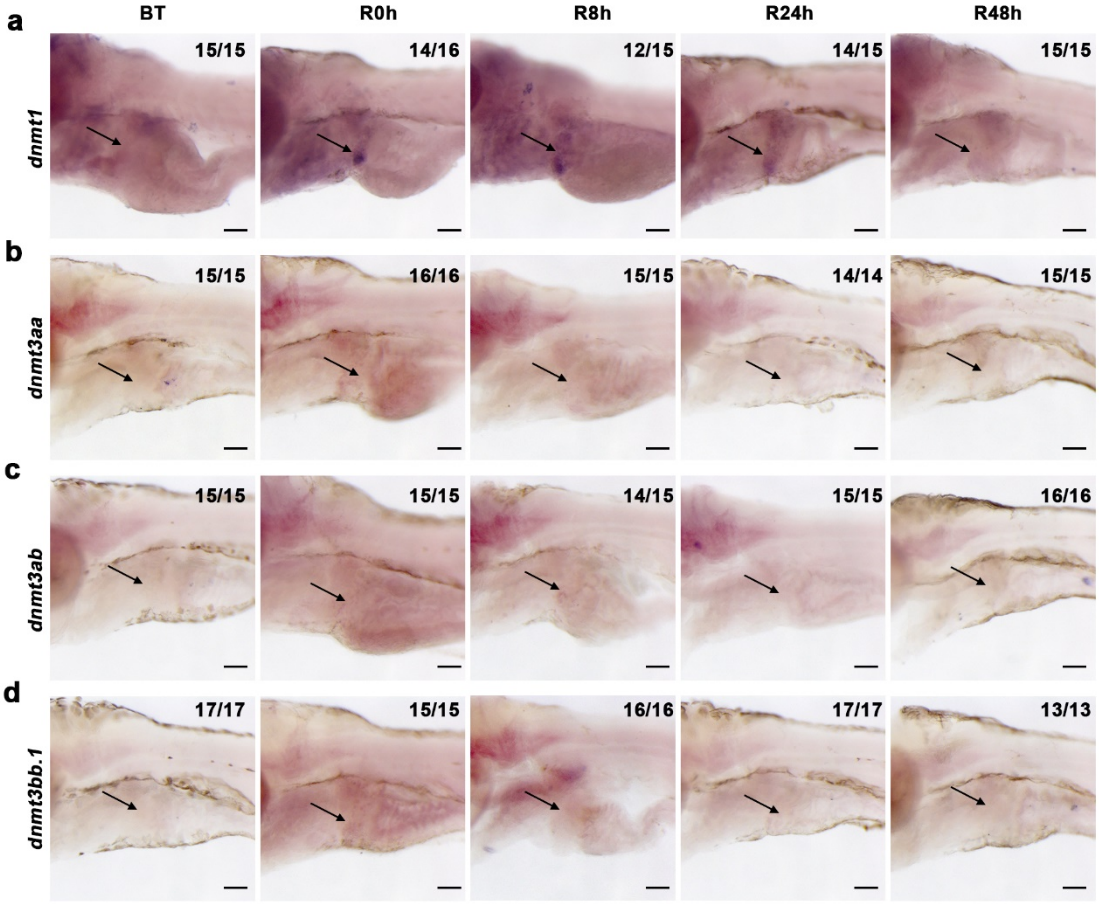

Supplementary Figure 1. *Dnmt1* is specifically upregulated in regenerating livers from R0h to R24h.

a-d WISH images showing the expressions of *dnmt1*, *dnmt3aa*, *dnmt3ab*, and *dnmt3bb.1* during liver regeneration from BT to R48h using the transgenic line *Tg(lfabp:Dendra2-NTR)*. Arrows indicate the liver regions. Numbers indicate the proportion of larvae exhibiting the expression shown. Scale bars: 100 µm. Abbreviation: BT, before treatment; R: regeneration time after the withdrawal of Mtz.

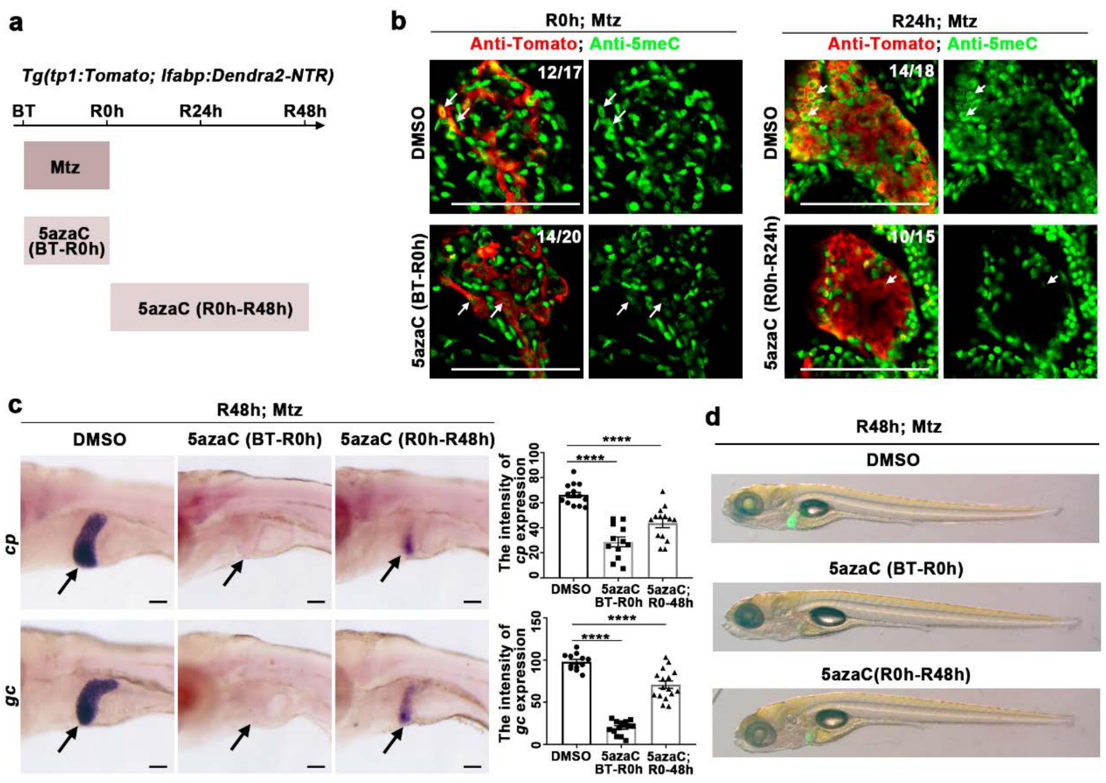

Supplementary Figure 2. DNA methylation inhibition reduces liver regeneration.

a Experimental scheme illustrating the 5azaC and Mtz treatment using the transgenic line *Tg(lfabp:Dendra2-NTR; tp1:Tomato)*. **b** Single-optical section images showing the expressions of 5meC and Tomato in 5azaC treatments and controls at R0h and R24h using the transgenic line *Tg(lfabp:Dendra2-NTR; tp1:Tomato)*. Note that the expressions of 5meC in Tomato positive BPPCs were reduced after 5azaC treatment. **c** WISH images showing the expressions of *cp* and *gc* in regenerating livers (arrows) in 5azaC treatments and controls. Quantification of the intensity of *cp* and *gc* expression in liver regions at R48h. Note that in 5azaC treated regenerating livers, the expressions of *cp* and *gc* were weak compared to controls at R48h. **d** The

phenotype images showing the body morphology of whole larvae at R48h after 5azaC and Mtz treatment and control. Asterisks indicate statistical significance: ****P <0.0001 using t tests analysis when compared to control. Numbers indicate the proportion of larvae exhibiting the expression shown. Scale bars: 50 µm in (b), 100 µm in (c); error bars, ±SEM. Abbreviation: BT, before treatment; R, regeneration time after the withdrawal of Mtz.

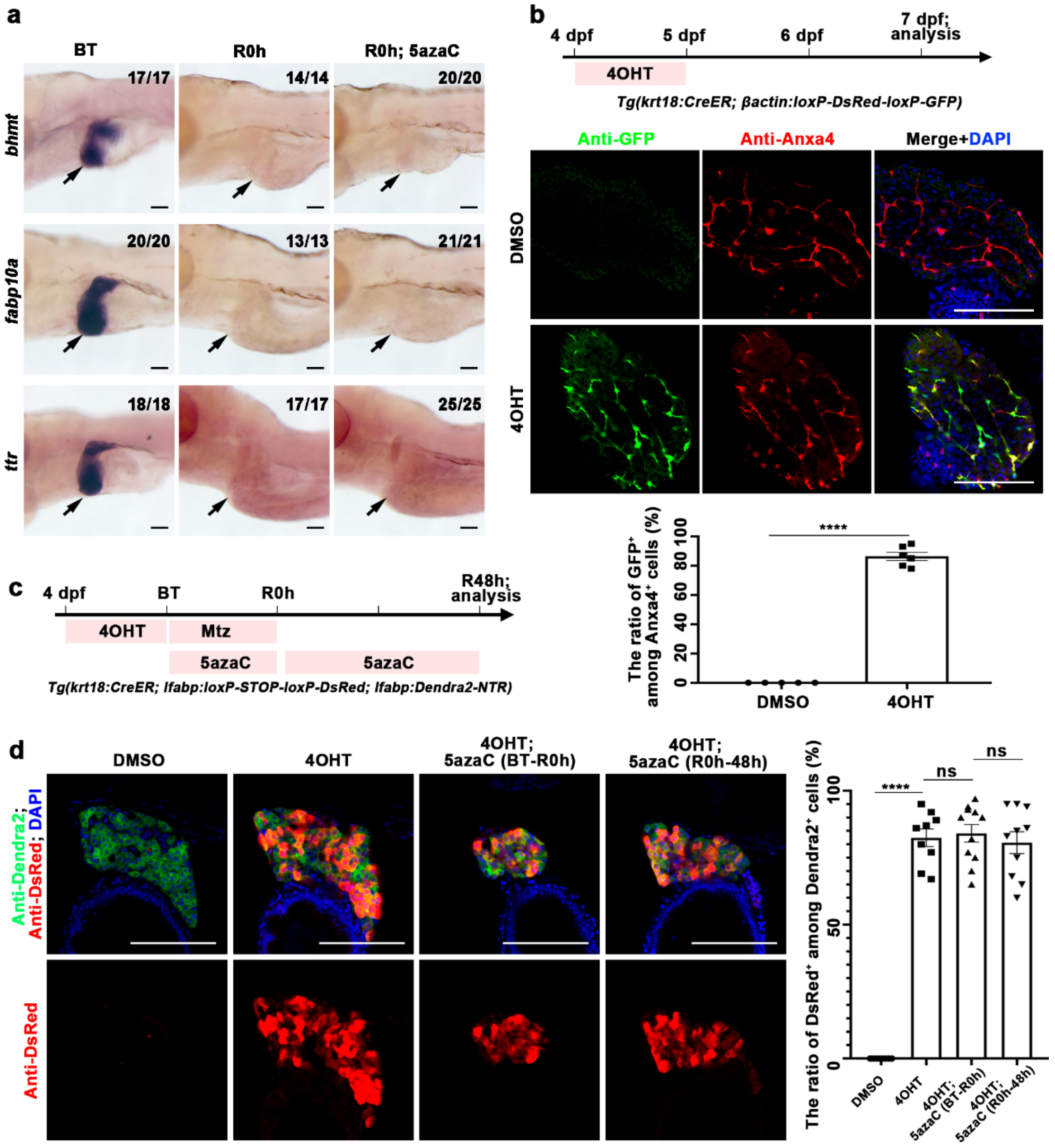

Supplementary Figure 3. New regenerating hepatocytes are derived from BECs after extreme hepatocyte ablation with 5azaC treatment.

a WISH images showing the expressions of *bhmt, fabp10* and *ttr* in BT and R0h livers (arrows) after 5azaC treatments and controls. **b** Experimental scheme illustrating the 4OHT or DMSO treatment and analysis at 7 dpf in *Tg(Krt18:CreER; βactin:loxP-DsRed-loxP-GFP)*. Single-optical section images showing the expressions of GFP and Anxa4 in liver at 7 dpf. Quantification of

the recombination efficiency in the DMSO or 4OHT treatment. Note that the expression of GFP merged with Anxa4 after 4OHT treatment. **c** Experimental scheme illustrating the transgenic line *Tg(Krt18:CreER; lfabp:loxP-STOP-loxP-DsRed; lfabp:Dendra2-NTR)* treated with 4OHT or DMSO, then analysis at R48h after Mtz and 5azaC treatment. **d** Single-optical section images showing the expressions of DsRed and Dendra2 in regenerating livers at R48h. Quantification of the percentage of BECs contribution in the DMSO or 4OHT treatment. Asterisks indicate statistical significance: ****P <0.0001 using t tests analysis when compared to control. Numbers indicate the proportion of larvae exhibiting the expression shown. Scale bars: 100 µm; error bars, ±SEM. Abbreviation: 4OHT, tamoxifen; dpf, days post fertilization; BT, before treatment; R: regeneration time after the withdrawal of Mtz; DAPI, 4', 6-diamidino-2-phenylindole; ns, no significant difference.

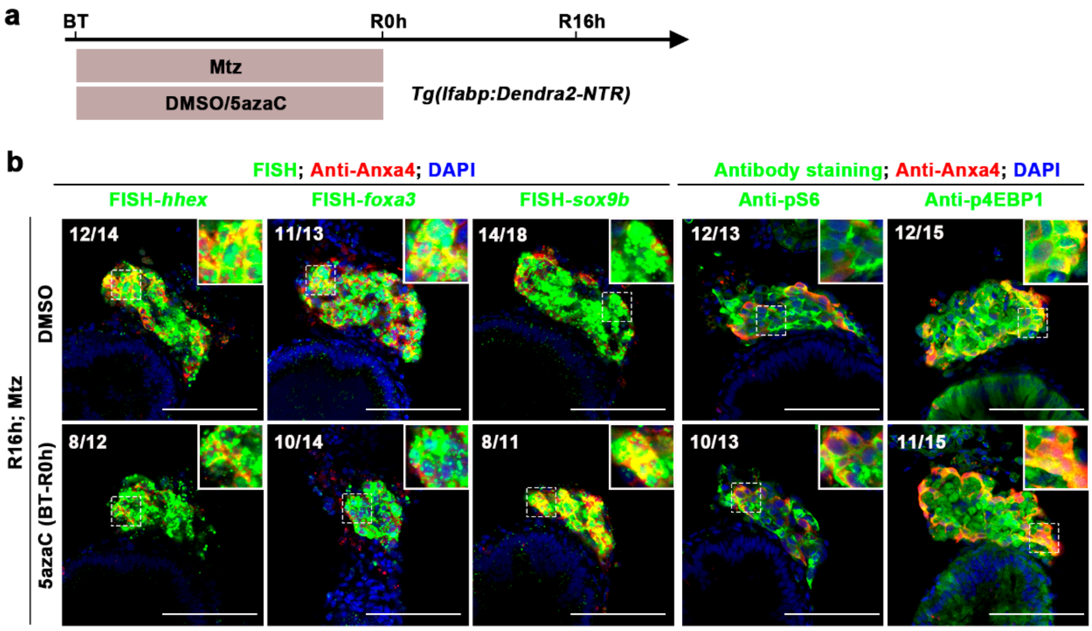

Supplementary Figure 4. Early DNA methylation inhibition delays the dedifferentiation of BECs.

a Experimental scheme illustrating the 5azaC treatment from BT-R0h (early DNA methylation inhibition) and analysis at R16h using the *Tg(lfabp:Dendra2-NTR)* transgenic line. **b** FISH and antibody staining images showing the expressions of *hhex*, *foxa3*, *sox9b*, pS6, p4EBP1, and Anxa4 at R16h after early DNA methylation inhibition. Numbers indicate the proportion of larvae exhibiting the expression shown. Scale bars: 100 µm. Abbreviations: BT, before treatment; R: regeneration time after the withdrawal of Mtz; FISH, fluorescent *in situ* hybridization; DAPI, 4', 6-diamidino-2-phenylindole.

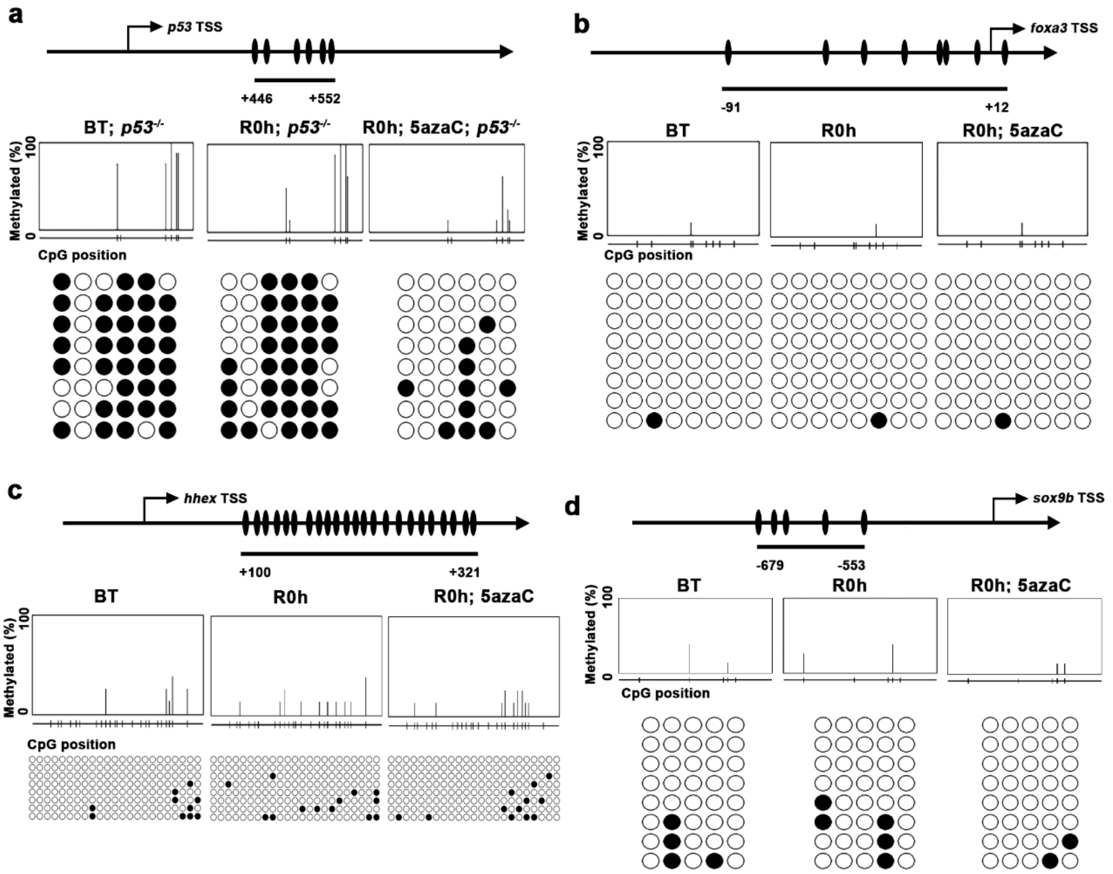

Supplementary Figure 5. Bisulfite sequencing analysis of DNA methylation of critical genes.

a Bisulfite sequencing analysis of DNA methylation at the *p53* locus in *p53* mutant liver treated with DMSO or 5azaC at BT and R0h. The region used for bisulfite sequencing spans +446 bp to +552 bp. **b** Bisulfite sequencing analysis DNA methylation at the *foxa3* locus in liver treated with DMSO or 5azaC at BT and R0h. The region used for bisulfite sequencing spans -91 bp to +12 bp. **c** Bisulfite sequencing analysis of DNA methylation at the *hhex* locus in liver treated with DMSO or 5azaC at BT and R0h. The region used for bisulfite sequencing spans +100 bp to +321 bp. **d** Bisulfite sequencing analysis of DNA methylation at the *sox9b* locus in liver treated with DMSO or

5azaC at BT and R0h. The region used for bisulfite sequencing spans -679 bp to -553 bp. Abbreviations: BT, before treatment; R: regeneration time after the withdrawal of Mtz; TSS, Transcription Start Site.

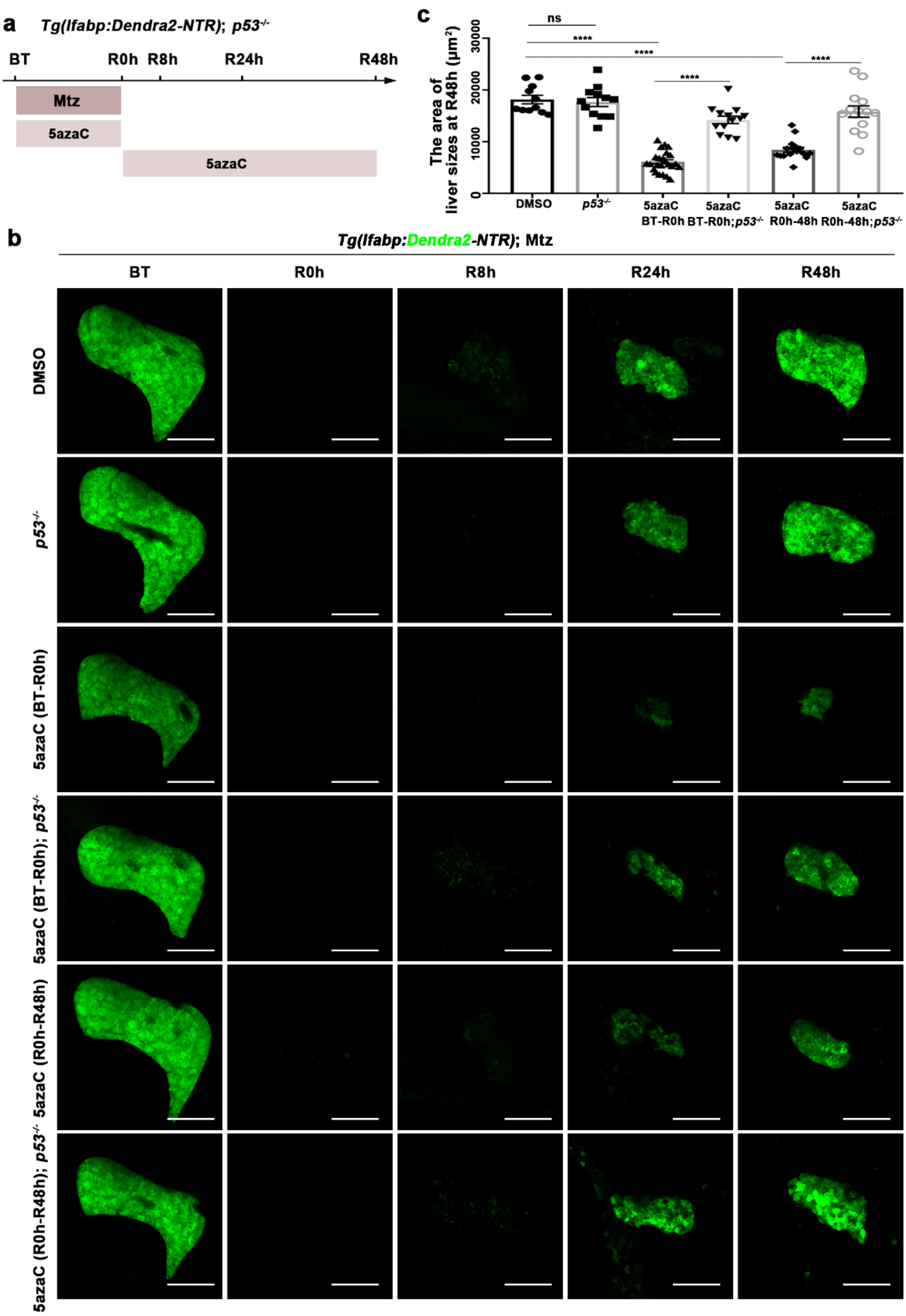

Supplementary Figure 6. Loss of *p53* partially rescues the liver regeneration defects after DNA methylation inhibitions.

a Experimental scheme illustrating the 5azaC treatment from BT-R0h (early DNA methylation inhibition) and R0h-R48h (late DNA methylation inhibition) and analyzing liver regeneration using the *p53* mutant and WT. **b** Confocal projection images showing the regeneration livers from BT to R48h after 5azaC treatment from BT-R0h or R0h-R48h in *p53* mutants and controls. Note that the liver regeneration defects caused by 5azaC treatment are partially rescued in *p53* mutant, and loss of *p53* cannot affect liver regeneration. **c** Quantification of the area of liver sizes at R48h. Asterisks indicate statistical significance: ****P <0.0001 using t tests analysis when compared to control. Scale bars: 100 µm; error bars, ±SEM. Abbreviation: BT, before treatment; R: regeneration time after the withdrawal of Mtz; ns, no significant difference.

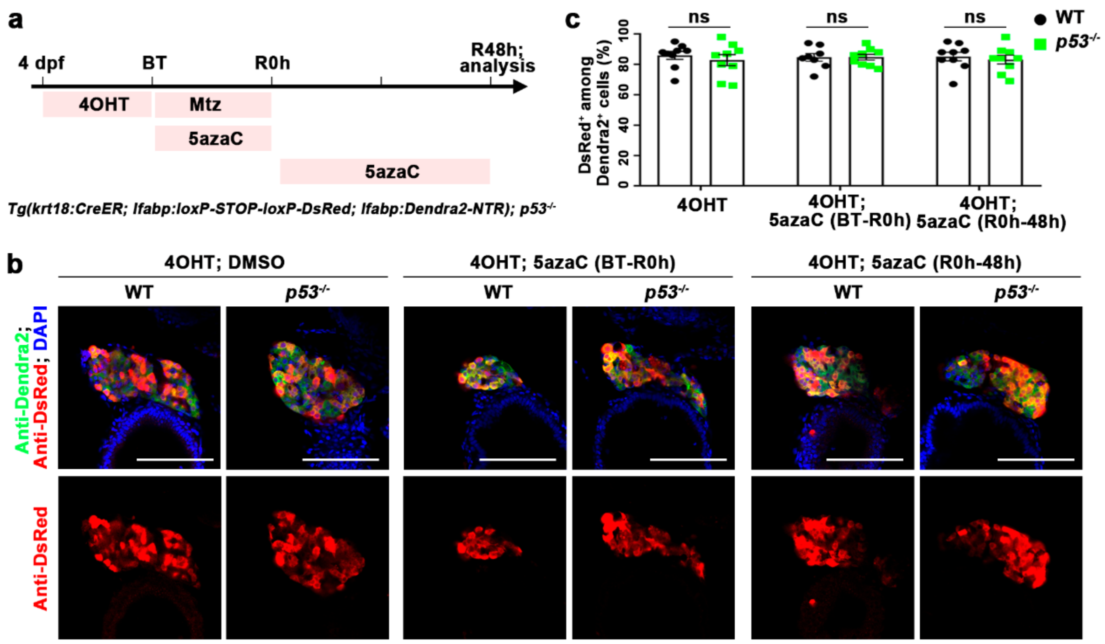

Supplementary Figure 7. New hepatocytes are derived from BECs after extreme hepatocyte ablation treated with 5azaC in *p53* mutant.

a Experimental scheme illustrating the transgenic line *Tg(Krt18:CreER; lfabp:loxP-STOP-loxP-DsRed; lfabp:Dendra2-NTR)* treated with 4OHT or DMSO, then analysis at R48h after Mtz and 5azaC treatment in *p53* mutant. **b** Single-optical section images showing the expression of DsRed and Dendra2 in regenerating livers at R48h. **c** Quantification of the percentage of Dsred+ cells among Dendra2+ cells. Statistics: using t tests analysis when compared to control. Scale bars: 100 μm; error bars, ±SEM. Abbreviations: 4OHT, tamoxifen; dpf, days post fertilization; BT, before treatment; R: regeneration time after the withdrawal of Mtz; DAPI, 4', 6-diamidino-2-phenylindole; ns, no significant difference.

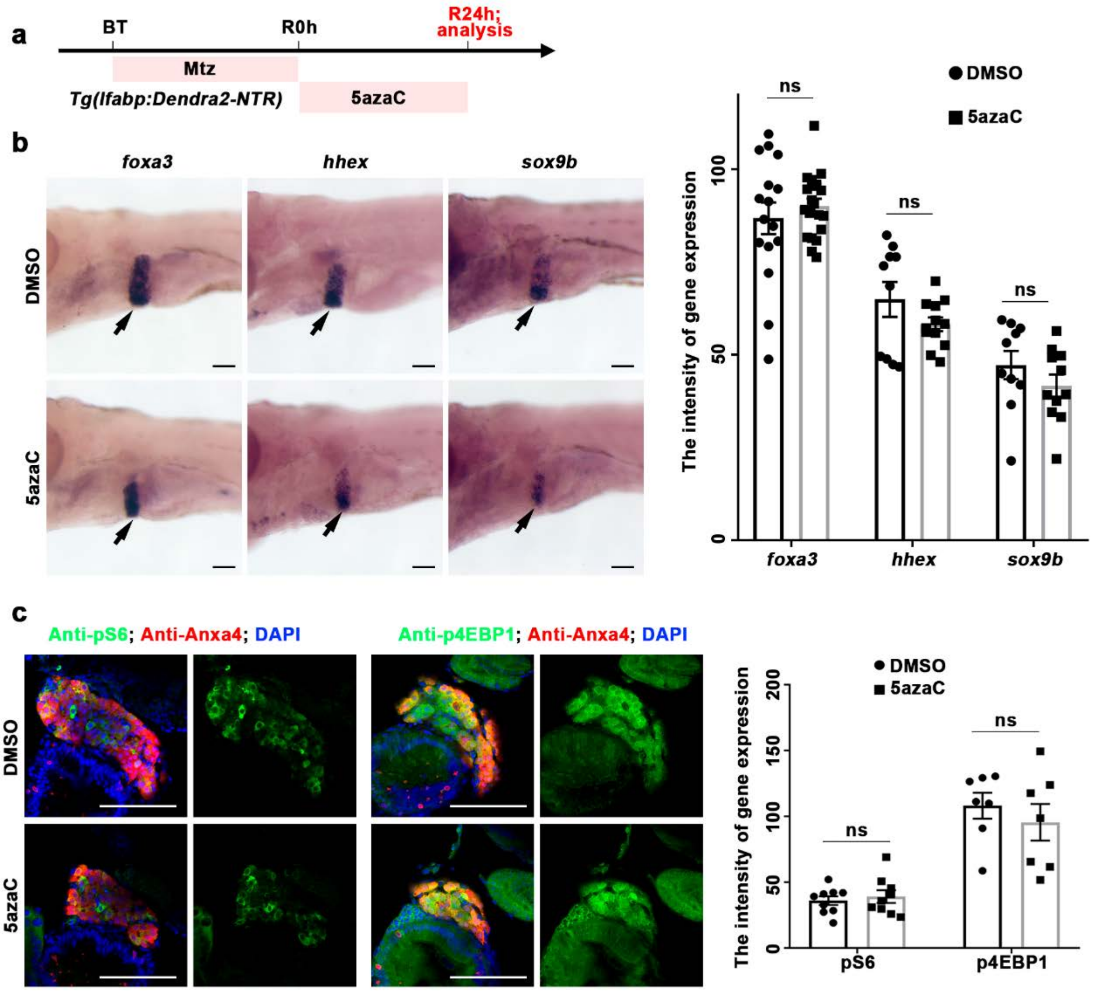

Supplementary Figure 8. Late DNA methylation inhibition cannot affect the dedifferentiation of BECs.

a Experimental scheme illustrating the 5azaC treatment from R0h-R24h (late DNA methylation inhibition) and analysis at R24h using the *Tg(lfabp:Dendra2-NTR)* transgenic line. **b** WISH images showing the expressions of *foxa3, hhex*, and *sox9b* in R24h livers (arrows) after late DNA methylation inhibition. Quantification of the intensity of *foxa3, hhex,* and *sox9b* expressions in liver regions at R24h. **c** Single-optical section images showing the expressions of pS6, p4EBP1, and Anxa4 at R24h after late DNA methylation inhibition.

Quantification of the intensity of pS6 and p4EBP1 expressions in liver regions at R24h. Statistics: using t tests analysis when compared to control. Scale bars: 100 µm; error bars, ±SEM. Abbreviations: BT, before treatment; R: regeneration time after the withdrawal of Mtz; ns, no significant difference; DAPI, 4', 6-diamidino-2-phenylindole.

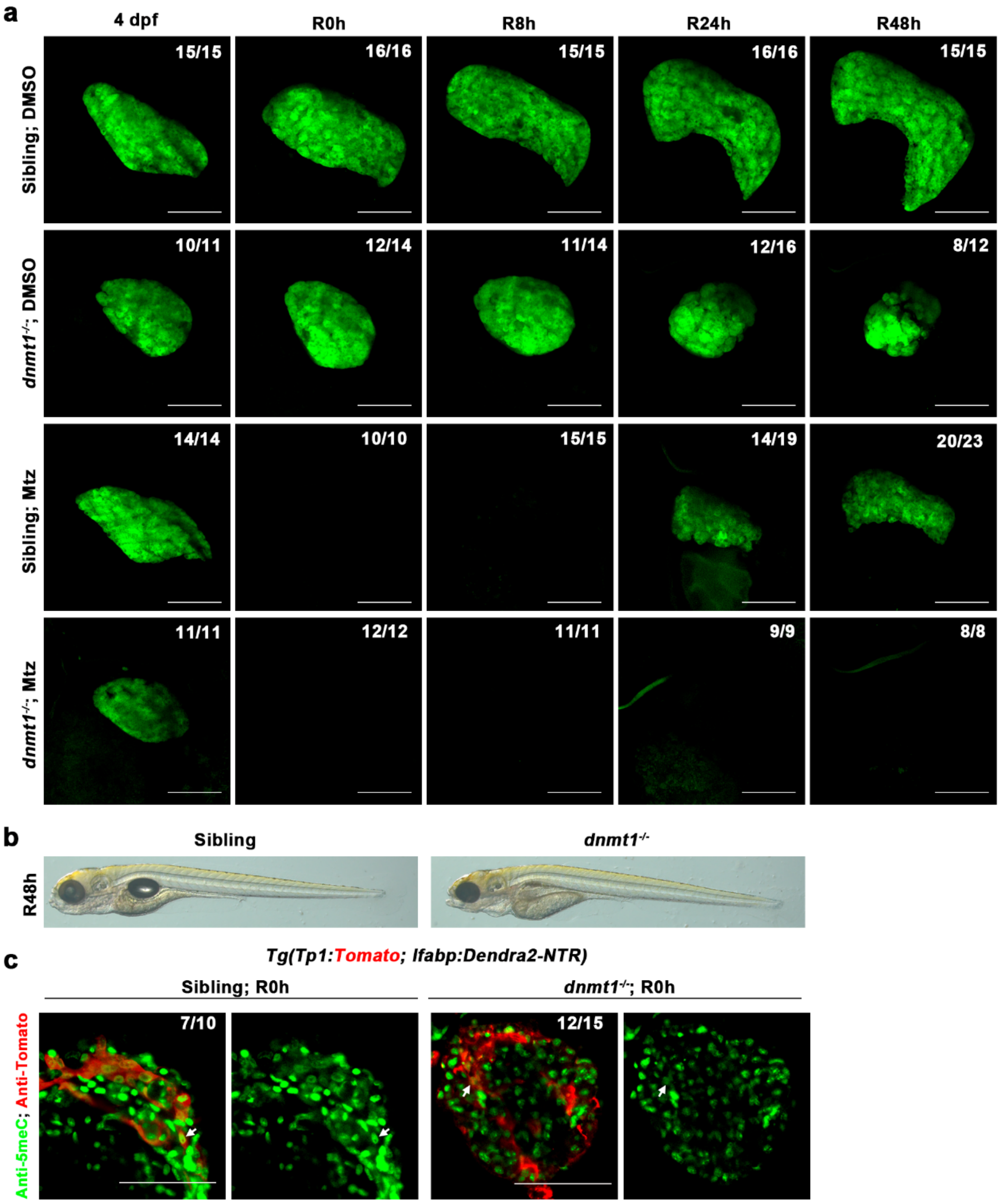

Supplementary Figure 9. Liver regeneration defects in *dnmt1* mutant.

a Confocal images showing the liver development and regeneration from 4 dpf to R48h in *dnmt1* mutant and sibling. Note that liver regeneration is blocked in *dnmt1* mutant. **b** The phenotype images showing the body morphology of whole larvae at R48h in *dnmt1* mutant and sibling. Note that

the *dnmt1* mutant has small eyes and an abnormal swim bladder. **c** Single-optical section images showing the expressions of 5meC and Tomato at R0h in *dnmt1* mutant and sibling using the transgenic line *Tg(lfabp:Dendra2-NTR; tp1:Tomato)*. Note that the expressions of 5meC in Tomato positive cells were reduced in *dnmt1* mutant compared to the sibling. Numbers indicate the proportion of larvae exhibiting the expression shown. Scale bars: 100 µm in (a), 50 µm in (c)**.** Abbreviation: dpf, days post fertilization; R, regeneration time after the withdrawal of Mtz.

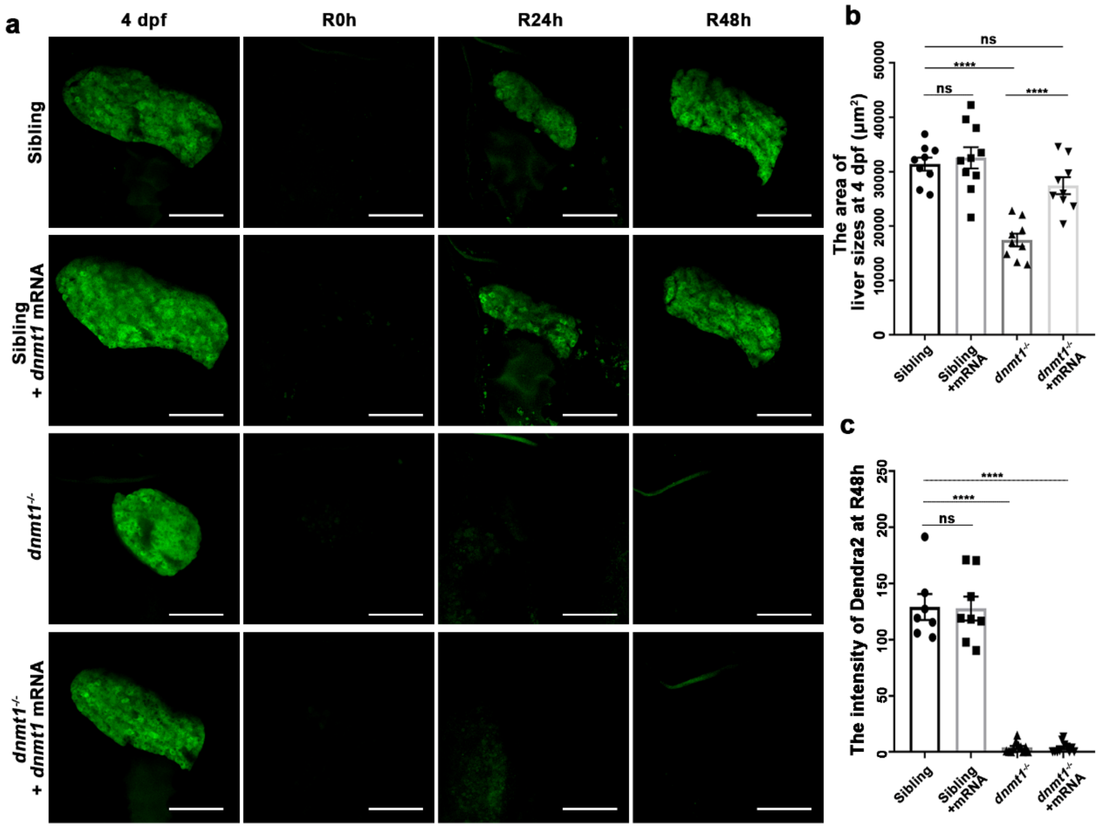

Supplementary Figure 10. Liver regeneration is still compromised in *dnmt1* mutant whose liver development defect was rescued by *dnmt1* mRNA.

a Confocal images showing the liver development and regeneration from 4 dpf to R48h in *dnmt1* mutant, mRNA-rescued *dnmt1* mutant, and their sibling using the transgenic line *Tg(lfabp:Dendra2-NTR)*. Note that liver regeneration is blocked both in *dnmt1* mutant and mRNA-rescued *dnmt1* mutant. **b** Quantification of the area of liver sizes at 4 dpf. **c** Quantification of the intensity of Dendra2 expression at R48h. Asterisks indicate statistical significance: ****P <0.0001 using t tests analysis when compared to control. Scale bars: 100 μm; error bars, ±SEM. Abbreviation: dpf, days post

fertilization; R: regeneration time after the withdrawal of Mtz; ns, no significant difference.

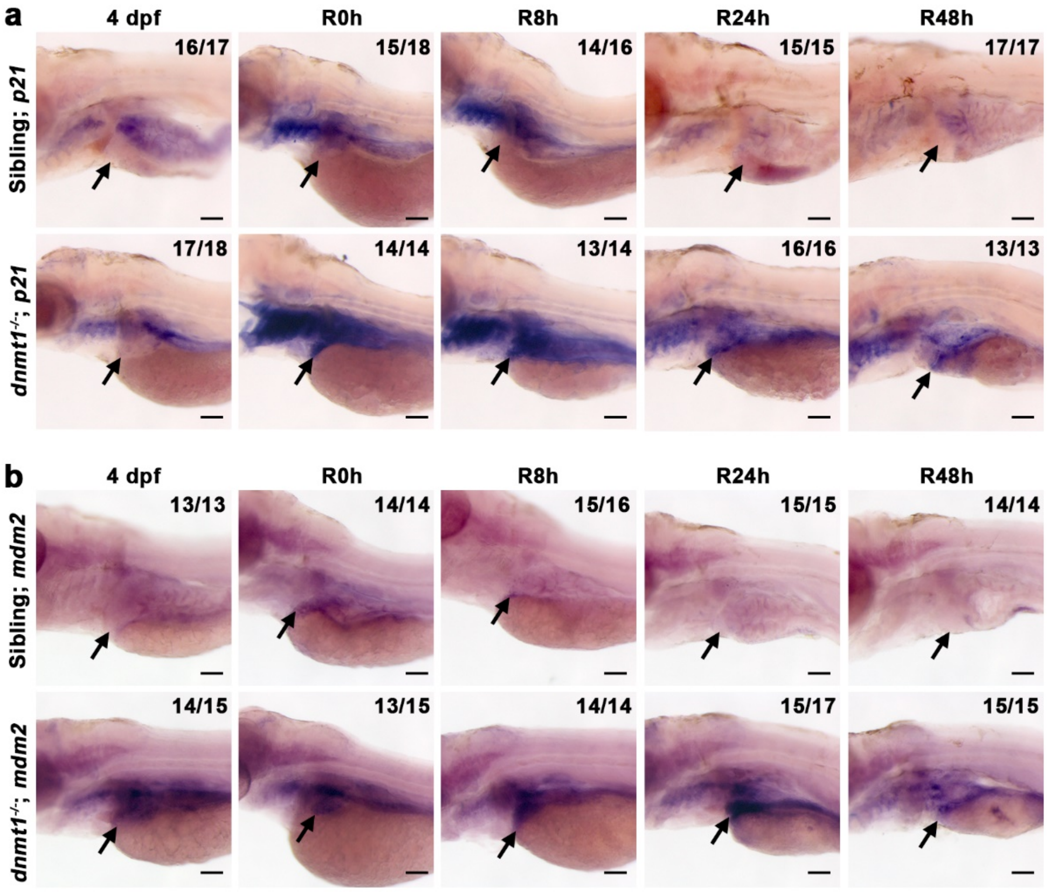

Supplementary Figure 11. The expressions of *p53* target genes *p21* and *mdm2* are significantly upregulated in *dnmt1* mutant livers after liver injury.

a WISH images showing the expressions of *p21* during liver regeneration from 4 dpf to R48h using the transgenic line *Tg(lfabp:Dendra2-NTR)*. **b** WISH images showing the expressions of *mdm2* during liver regeneration from 4 dpf to R48h using the transgenic line *Tg(lfabp:Dendra2-NTR)*. Numbers indicate the proportion of larvae exhibiting the expression shown. Scale bars: 100 µm. Abbreviation: dpf, days post fertilization; R: regeneration time after the withdrawal of Mtz.

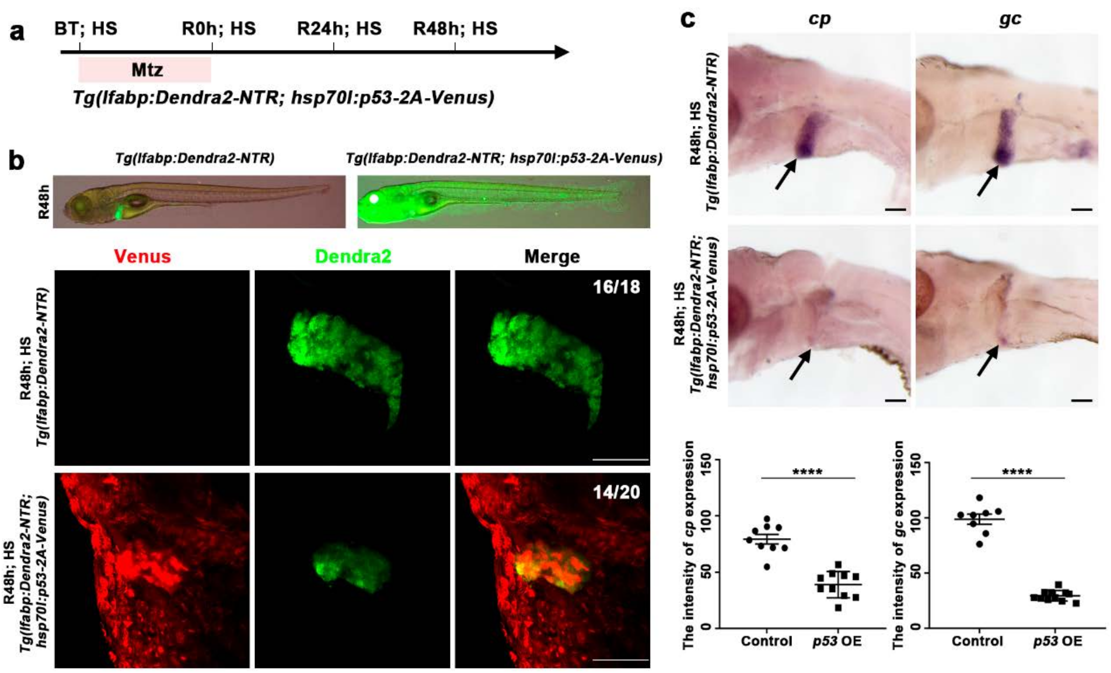

Supplementary Figure 12. *p53* activation reduces BECs-mediated liver regeneration.

a Experimental scheme illustrating the time of heat shock (HS) in the transgenic line *Tg(lfabp:Dendra2-NTR; hsp70l:p53-2A-Venus)*. **b** Confocal images showing the expressions of Venus and Dendra2 in regeneration livers at R48h. The phenotype images showing the body morphology of whole larvae at R48h after heat shock. **c** WISH images showing the expressions of *gc* and *cp* at R48h after heat shock. Quantification of the intensity of *cp* and *gc* expression in liver regions at R48h. Note that the expressions of *gc* and *cp* were decreased in the *p53* overexpression transgenic line. Asterisks indicate statistical significance: ****P <0.0001 using t tests analysis when compared to control. Numbers indicate the proportion of larvae exhibiting the expression shown. Scale bars: 100 µm; error bars, ±SEM. Abbreviation: BT, before treatment; R: regeneration time after the withdrawal of Mtz; HS, heat shock;

OE, overexpression.

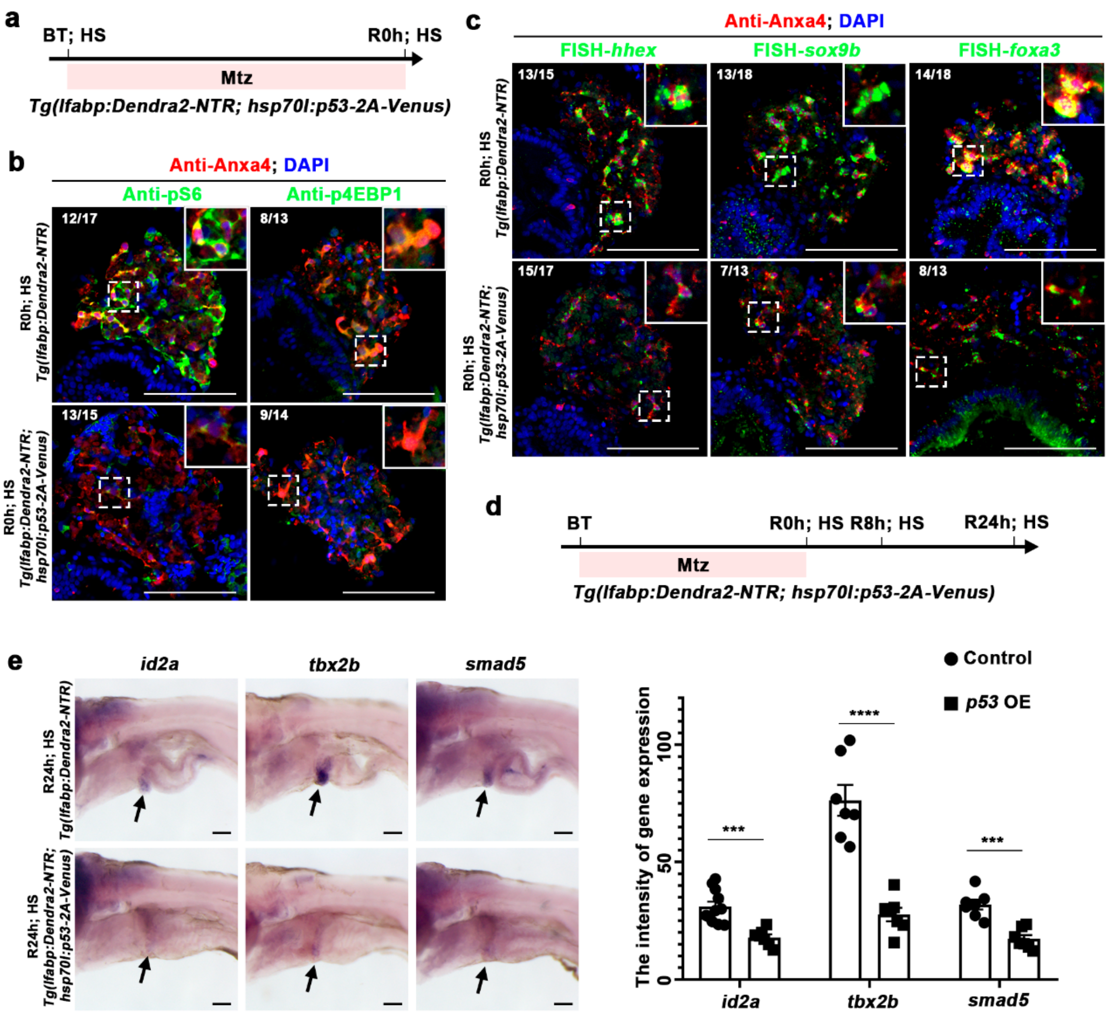

Supplementary Figure 13. ***p53*** **overexpression reduces mTORC1 and Bmp signaling during liver regeneration.**

a Experimental scheme illustrating the Mtz treatment and heat shock with *Tg(lfabp:Dendra2-NTR; hsp70l:p53-2A-Venus)* transgenic line. **b** Single-optical section images showing the expressions of pS6, p4EBP1, and Anxa4 at R0h after heat shock and Mtz treatment. **c** FISH and antibody staining images showing the expressions of *hhex*, *foxa3*, *sox9b*, and Anxa4 at R0h after Mtz treatment and heat shock. **d** Experimental scheme illustrating the Mtz treatment and heat shock with *Tg(lfabp:Dendra2-NTR; hsp70l:p53-2A-Venus)* transgenic line. **e** WISH images showing the expressions of *id2a,*

tbx2b, and *smad5* at R24h, in regenerating livers (arrows) after Mtz treatment and heat shock. Quantification of the intensity of *id2a, tbx2b,* and *smad5* expression in liver regions at R24h. Asterisks indicate statistical significance: ***P<0.001; ****P<0.0001 using t tests analysis when compared to control. Numbers indicate the proportion of larvae exhibiting the expression shown. Scale bars: 100 µm; error bars, ±SEM. Abbreviations: BT, before treatment; R: regeneration time after the withdrawal of Mtz; HS, heat shock; OE, overexpression; FISH, fluorescent *in situ* hybridization; DAPI, 4', 6-diamidino-2-phenylindole.

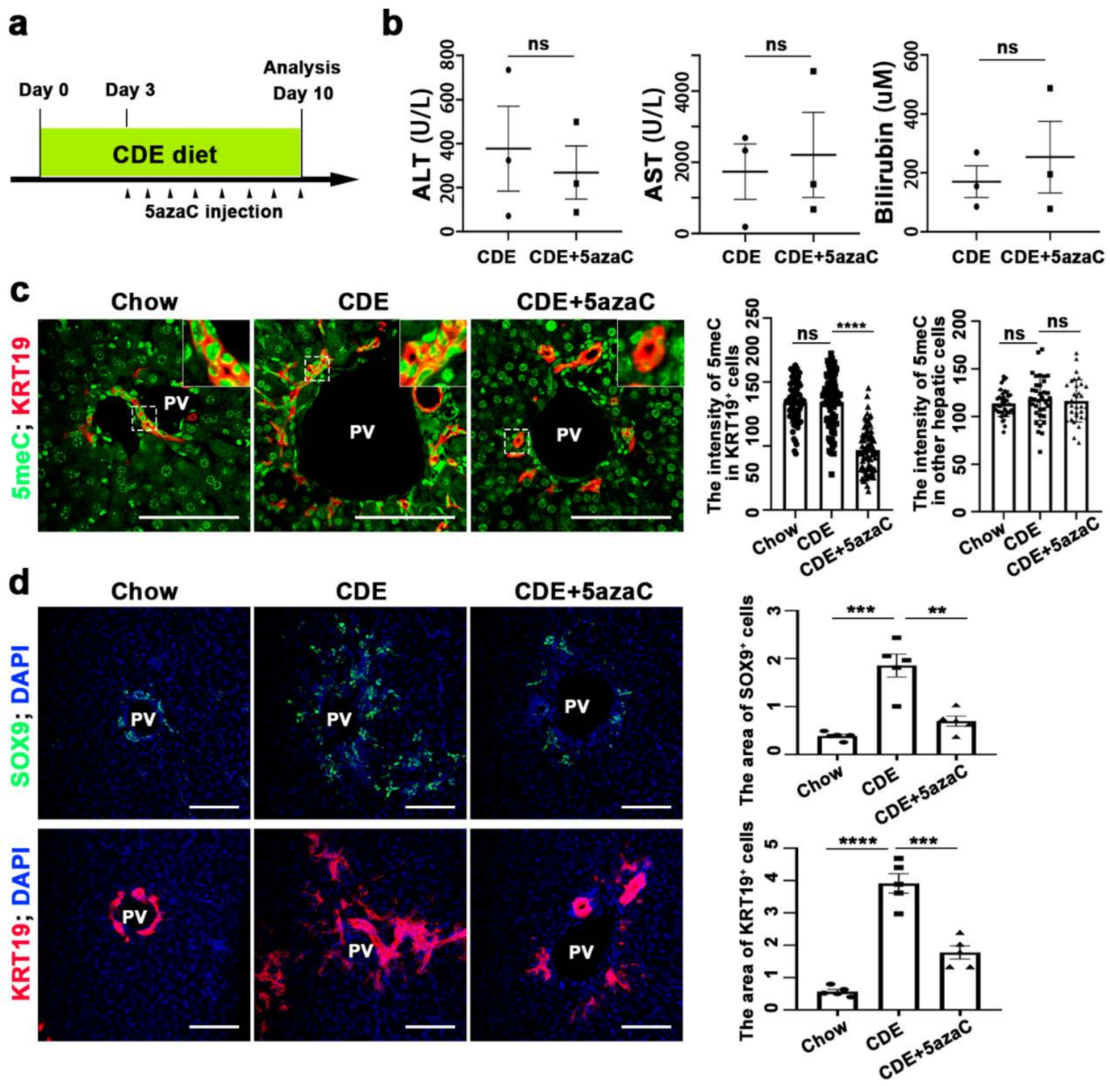

Supplementary Figure 14. DNA methylation inhibition impairs hepatic progenitor cells activation in mice fed a CDE diet.

a Experimental scheme illustrating the period of a CDE diet and 5azaC injection stages. **b** Quantification of the levels of ALT, AST, and bilirubin in serum (n=3). **c** Epifuorescence images showing the KRT19 and 5meC staining in liver sections and their quantifications; for KRT19+ cells from 5 biological replicates (n=71, Chow; n=64, CDE; n=81, CDE+5azaC); for other hepatic cells from 5 biological replicates (n=37, Chow; n=35, CDE; n=32, CDE+5azaC). **d** Epifuorescence images showing the KRT19 and SOX9

staining in liver sections and their quantifications (n=5 biological replicates for each group). Asterisks indicate statistical significance: **$P<0.01$; ***$P<0.001$; ****$P<0.0001$ using t tests analysis when compared to control. Scale bars: 100 µm; error bars, ±SEM. Abbreviation: DAPI, 4',6-diamidino-2-phenylindole; CDE, choline-deficient, ethionine-supplemented diet; ns, no significant difference.

Supplementary Tables

Supplementary Table 1. List of fish strains used in this study

Fish strain	Function	Source
Tg(lfabp:Dendra2-NTR)^{cq1}	Hepatocyte-specific ablation	(He et al., 2014)[1]
Tg(Tp1:Tomato)^{cq109}	Tomato fluorescent protein under the control of an element containing 12 RBP-Jκ binding sites marks BECs	This paper
p53^{M214K}	*p53* mutant	(Berghmans et al., 2005)[2]
Tg(hsp70l:Bmp2b)^{fr13}	Bmp2b overexpression via heat-shock	(Shin et al., 2007)[3]
dnmt1^{s872}	*dnmt1* mutant	(Anderson et al., 2009)[4]
Tg(krt18:tet3G; tre3G:p53-2A-Venus)	*p53* overexpression induced by Tet3G protein and doxycycline treatment in BECs	This paper
Tg(Tp1:eGFP)^{um14}	GFP fluorescent protein under the control of an element containing 12 RBP-Jκ binding sites marks BECs	(Parsons et al., 2009)[5]
Tg(krt18:creER^{cq74}*; βactin:loxP-DsRed-loxP-GFP*^{s928}*)*	Cre/loxP lineage tracing system induced by CreER and 4OHT in BECs	(Kikuchi et al., 2010),[6] (He et al., 2019)[7]
Tg(krt18:creER^{cq74}*, lfabp:loxP-STOP-loxP-DsRed*^{cq4}*)*	Cre/loxP lineage tracing system induced by CreER and 4OHT in hepatocyte	(He et al., 2014),[1] (He et al., 2019)[7]
Tg(hsp70l:p53-2A-Venus)	Whole body *p53* overexpression via heat-shock	This paper

Supplementary Table 2. List of oligonucleotides used in this study

Primer name	sequence
Whole-mount *in situ* hybridization (WISH) and Fluorescence *in situ* hybridization (FISH) primers	
*cp*_F	CTGCGGAGGAGGACGACACGG
*cp*_R	ATTGTAATACGACTCACTATAGGGGGCTGACGGTGTCGACATGC
*gc*_F	CCTCCAAGTCATTGGAATTG
*gc*_R	ATTGTAATACGACTCACTATAGGGCGGAATGGGTACGACTGGAC
*dnmt1*_F	AGCCACAGTCCTGGTTCCAG
*dnmt1*_R	ATTGTAATACGACTCACTATAGGGGGAACAGCATTACCAACTTG
*dnmt3aa*_F	GGAGACATCAGGAACATCACAC
*dnmt3aa*_R	ATTGTAATACGACTCACTATAGGGGGACACGTCAGTGTAATGGACG
*dnmt3ab*_F	CGTCCTGTCACTTTTTGATGGG
*dnmt3ab*_R	ATTGTAATACGACTCACTATAGGGGGAATTCGACCGAGTAGTGATG
*dnmt3bb.1*_F	CATTGCCACAGGTTATCTGGTG
*dnmt3bb.1*_R	ATTGTAATACGACTCACTATAGGGGGAAATGCTGGTCTTTGCCTTG
*hhex*_F	CGAACTCCTCTTTCACCAGCC
*hhex*_R	ATTGTAATACGACTCACTATAGGGCATAGGGTGAACTGATGCTCG
*foxa3*_F	CGAGCGCCATGAACTCAGTG
*foxa3*_R	ATTGTAATACGACTCACTATAGGGGTGCCCTTGGTGCTGCTGC
*sox9b*_F	GGGCTGAAGATGAGTGTGTC
*sox9b*_R	ATTGTAATACGACTCACTATAGGGGATGACATCACTGCTCAGCTC
*p53*_F	AAGAACAGCCTCAGCCATCC
*p53*_R	TAATACGACTCACTATAGGGTCCATTCAGCACCAAGCTGT
*p21*_F	CAAGCGGATCCTACGTTCAC
*p21*_R	TAATACGACTCACTATAGGGCTGGGGTTTTCTCCACTTCA
*mdm2*_F	GTAGGTATATCTCGCAGTGAGG
*mdm2*_R	TAATACGACTCACTATAGGGCTGTGATGATGTGGTCTGAGAG
*id2a*_F	CCTGCTGTCAACATGAAGGC
*id2a*_R	ATTTAGGTGACACTATAGAGGTAAAGTGTCCTGCTGTCC

*tbx2b*_F	GGATGCACCGATGAGAGATCC
*tbx2b*_R	ATTTAGGTGACACTATAGACCAGGTGAATGACCAGCATC
*smad5*_F	GTGCAGCCCAGCACTATGAC
*smad5*_R	ATTTAGGTGACACTATAGACTCTGCATACACCTCTCCTC
*bhmt*_F	GACCTGCTGATCGCTGAGTAC
*bhmt*_R	TAATACGACTCACTATAGGGGTCTCAGTGTTTAGCGTCCG
*fabp10a*_F	CTCCAGAAAGCATGGCCTTC
*fabp10a*_R	TAATACGACTCACTATAGGGGAGTTATGGTGAAACGCTTCAG
*ttr*_F	CAGTTCTGCTCGAACATGGC
*ttr*_R	TAATACGACTCACTATAGGGGATGTCAGTCATGTGCCTTG
qPCR primers	
*eef1a1l1*_F	CTGGAGGCCAGCTCAAACAT
*eef1a1l1*_R	ATCAAGAAGAGTAGTACCGCTAGCATTAC
*p53*_F	CTCTCCCACCAACATCCACT
*p53*_R	GATTGCCCTCCACTCTTATCA
Methylation PCR primers	
*p53*_F	GGTGGTTTTTGTTGGGATATTATTA
*p53*_R	AAAACCTAAACCTAAATCCATAATC
*foxa3*_F	TTGAGAAGTTTAAAGTGAAGTTGTG
*foxa3*_R	CAAAACAACAAAAAACAAAAAAAAC
*hhex*_F	TTATATAGAAGATATTTTGGGAAGAAT
*hhex*_R	CATTTAAACAAAAAAAACAAATTAC
*sox9b*_F	TTTGTGAAGTTAAGTTAGATTAAGAG
*sox9b*_R	CAAACATACACTCATTTAAAAAAAA
dnmt1 CDS primers	
*dnmt1-cds*_F	TGCAGGATCCCATCGATTCGAATTCATGCCTACCAAGACCTCATTGTC
*dnmt1-cds*_R	TAGAGGCTCGAGAGGCCTTGAATTCTTAGTCAGAGAGCTCCATTTTCTCC

Underlined are T7, Sp6, or homologous arm primer sequences.

Supplementary References

1. He, J., Lu, H., Zou, Q. & Luo, L. Regeneration of liver after extreme hepatocyte loss occurs mainly via biliary transdifferentiation in zebrafish. *Gastroenterology* **146**, 789-800 e788 (2014).
2. Berghmans, S., *et al.* tp53 mutant zebrafish develop malignant peripheral nerve sheath tumors. *Proc Natl Acad Sci U S A* **102**, 407-412 (2005).
3. Shin, D., *et al.* Bmp and Fgf signaling are essential for liver specification in zebrafish. *Development* **134**, 2041-2050 (2007).
4. Anderson, R.M., *et al.* Loss of Dnmt1 catalytic activity reveals multiple roles for DNA methylation during pancreas development and regeneration. *Dev Biol* **334**, 213-223 (2009).
5. Parsons, M.J., *et al.* Notch-responsive cells initiate the secondary transition in larval zebrafish pancreas. *Mech Dev* **126**, 898-912 (2009).
6. Kikuchi, K., *et al.* Primary contribution to zebrafish heart regeneration by gata4(+) cardiomyocytes. *Nature* **464**, 601-605 (2010).
7. He, J., *et al.* Mammalian Target of Rapamycin Complex 1 Signaling Is Required for the Dedifferentiation From Biliary Cell to Bipotential Progenitor Cell in Zebrafish Liver Regeneration. *Hepatology* **70**, 2092-2106 (2019).
